# Thermal, economic, and environmental assessment of optimal aerogel insulation thickness compared with conventional materials in syrian climates

**DOI:** 10.1038/s41598-026-45028-9

**Published:** 2026-03-24

**Authors:** Lujain Dory, Ibrahim Alghoraibi, Nada Altunji, Wassim Abdelwahed

**Affiliations:** 1 Department of Environmental Technologies Engineering, Faculty of Technical Engineering, Aleppo University, Aleppo, Syria; 2 Department of physics, Faculty of science, Damascus University, Damascus, Syria; 3 Department of basic and supporting sciences, Faculty of Pharmacy, Arab International University, Ghabaghib, Dara, Syria; 4 Scientific Expert and Consultant, Consultant Expert – Scientific Affairs and Research Council – Al-Sham Private University, Al Tal, Syria; 5 Department of Pharmaceutical Technology, Faculty of pharmacy, Aleppo University, Aleppo, Syria

**Keywords:** Life-cycle cost analysis (LCC), Heating and cooling degree-days (HDD/CDD), Aerogel insulation, Energy efficiency in buildings, Optimal insulation thickness, Conventional insulation materials, Economic feasibility, Climatic zone analysis, Climate sciences, Energy science and technology, Engineering, Environmental sciences

## Abstract

The current study aims to optimize the use of insulation materials by determining the optimal thickness for exterior building walls across various Syrian climatic zones and different energy sources. The study focuses on a comparative analysis between nano-aerogel and conventional insulation materials, including Extruded Polystyrene (XPS), Polyurethane (PUR), and Glass Wool (GW), utilizing Heating and Cooling Degree-Days (HDD/CDD) methodology, to enhance energy efficiency in buildings. An integrated assessment was conducted through Life-Cycle Cost Analysis (LCC), encompassing thermal and environmental aspects, with a competitive advantage featuring the integration of space-saving requirements and the calculation of economic feasibility resulting from the rental of floor areas recovered through the use of low-thickness aerogel insulation. The results indicated that Glass Wool (GW) emerged as a highly feasible option prior to integrating space savings, with thicknesses ranging from 0.06 to 0.15 m. In contrast, aerogel achieved the highest efficiency when space savings were incorporated into the analysis, particularly in cities with high rental values such as Damascus, Aleppo (diesel fuel), and Latakia (diesel fuel), due to its remarkably low optimal thickness (0.001–0.011 m). Specifically, aerogel reached its peak efficiency in the city of Latakia (diesel fuel) at an exceptionally low optimal thickness of 0.001 m. At this level, the reduction in initial capital expenditure allowed space-saving revenues to dominate the economic balance, sharply decreasing the discounted payback period to just 1.2 years (the shortest value recorded in this study). This accelerated cost recovery pace enabled aerogel to outperform all studied conventional materials. The study concludes that there is a critical trade-off requiring a precise balance between the material’s embodied energy and the sustainable economic and spatial gains, making the selection of insulation material and its optimal thickness a decision that depends primarily on the economic and spatial context of the project. Environmentally, conventional materials achieved higher emission reduction rates compared to aerogel due to their larger adopted optimal thicknesses for all study scenarios presented.

## Introduction

Anthropogenic environmental degradation and the steady rise of global greenhouse gas emissions are primarily driven by patterns of energy consumption. The building sector represents a major contributor to this challenge, accounting for nearly 40% of total global energy use annually, with a substantial portion dedicated to heating, cooling, and lighting. Consequently, the environmental burden associated with CO₂, NOₓ, and CFC emissions has intensified research and industrial efforts toward the development of sustainable heating and cooling solutions^1^. Over the past decade, international climate policies have significantly stimulated research in renewable energy^2–6^. Within this global framework, CO₂ emissions from the construction sector is essential, since this sector is responsible for approximately 45% of total global CO₂ emissions^7^, with profound implications for climate change^8,9^. In alignment with these international objectives, the European Union’s 2030 strategic plan targets a 40% reduction in greenhouse gas emissions relative to 1990 levels, alongside 27% improvement in energy efficiency^10^. The 2023 International Energy Agency (IEA) report further emphasizes the urgent need to halve operational emissions by 2030 to stay on track toward a net-zero trajectory^11^.

Research indicates that both operational and embodied energy contribute significantly to a building’s total energy use. Studies highlight a critical trade-off between them; reducing operational energy often requires the use of advanced insulation materials with higher embodied energy^12^. A comprehensive review by Chastas et al.^13^ of 90 Life-cycle energy assessment cases showed that embodied energy dominates in high-performance structures, reaching 26%–57% in low-energy buildings and 74%–100% in near-zero energy buildings (nZEBs). Given that the European Energy Performance of Buildings Directive (EPBD) mandated nZEB standards for new buildings, analyzing this trade-off is crucial for achieving Optimization in the building’s total energy use. Therefore, integrating sustainable strategies at early design stages to evaluate different material scenarios offers a vital opportunity to launch an Optimization in the trade-off between embodied versus operational energy^12^. In this context, the Optimization process is considered as an engineering tool to ensure that the initial environmental investment, stemming from the high embodied energy of certain materials, is effectively compensated for through substantial reductions in operational energy, ultimately achieving the lowest possible energy footprint throughout the building’s entire life cycle.

Within this context, thermal insulation is recognized as one of the most effective measures to reduce energy consumption in buildings^14–17^Consequently, selecting appropriate insulation materials and determining their optimal thickness have become a central focus of research^18^.. Applying thermal insulation significantly reduces heat loss, thereby minimizing energy consumption and environmental footprint^19,20^, making the selection of appropriate insulation materials and optimal thickness particularly vital^21^.

The pursuit of enhanced thermal performance may follow two primary strategies: increasing the thickness of conventional insulation materials, which reduce usable interior space, or employing advanced nano-based super-insulation materials such as aerogels, which provide ultra-low thermal conductivity with minimal thickness a major advantage in high-density urban environments^22^.

It is well established that the heat transmission decreases exponentially with increased insulation thickness, whereas costs rise linearly, creating a critical economic threshold. Beyond this point, further thickness yields diminishing returns. The optimal insulation thickne**ss** therefore represents the equilibrium point where the combined cost of insulation and energy is minimized while ensuring adequate thermal comfort^23^.

The optimization of insulation thickness is a well-established research field, with numerous studies employing life-cycle cost (LCC) analysis in combination with the degree-day (DD) method^24–29^. This approach provides a simple yet effective tool to estimate optimal parameters across various climates^1,24,25,30–34^.

Regional studies have demonstrated significant variations in optimal insulation requirements. For example, in Middle Eastern contexts, Alsayed et al.^27^analyzed eight Palestinian governorates and found polyurethane foam to be the most efficient among polystyrene, rigid polyurethane foam, and rock wool, with optimal thickness ranging from 0.004 to 0.09 m. Similarly, Idchabani et al^35^. reported notable variations in optimal thickness (3.4–16.8 cm) for expanded polystyrene, polyurethane, and cork in Morocco buildings, highlighting the significant influence of both insulating material and energy sources.

In another study, Dombayci et al^25^.. evaluated five energy sources and confirmed that fuel type critically influences economic optimization, identifying the coal-expanded polystyrene combination as the most economical, delivering 14 $/m² life-cycle savings over a 10-year period, while providing comparative performance benchmarks for rock wool insulation under identical energy source conditions.

Further environmental assessments, such as Kallioğlu’s research^36^ in Turkey, revealed that expanded polystyrene (EPS) achieved 73.06% energy reduction with a Payback Period of 1.25 years, and a corresponding 85% decrease in SO₂ emissions.

Subsequent studies (e.g., Shekarchian et al.)^37^validated these findings by quantifying the reduction in annual CO₂ and SO₂ emissions from insulation, underscoring its environmental value. Similarly, Fertelli et al^38^. examined multiple cities in Turkey and found that optimal thickness values varied between 0 and 0.179 m, depending on fuel type and wall composition confirming the strong correlation between climate, fuel, and insulation performance. Complementing these empirical findings, methodological innovations include the framework developed by Raza and Aggarwal^39^. Their comparative framework, which employed both degree-day and annual full-load cooling hours methods to evaluate three insulation materials in a brick-sandwich assembly, established Expanded Polystyrene (EPS) with LPG heating as the optimal insulation material.

Expanding to Asian contexts, Shahid et al..‘s cooling degree-day analysis^40^ across four major Indian cities evaluated five insulation materials - fiberglass rigid, urethane rigid, fiberglass urethane, perlite, and extruded polystyrene showing insulation thickness variations between 1 and 12 cm achieving potential energy savings of up to 80%.In specialized applications, Akyüz *et al.‘s* evaluation^22^ of thermal insulation at Istanbul’s Hasan Polatkan Airport terminal demonstrated 48–56% heat loss reduction with environmental Payback Periods shorter than economic Payback Periods.

Recent studies have also emphasized space efficiency as an additional economic variable. Ali et al^41^. introduced a novel framework integrating spatial efficiency in the evaluation of nano-materials, showing that aerogels not only save energy but also maximize usable floor area, leading to additional economic gains in high-value real estate. Despite extensive research on conventional insulation materials^27,35,36^, a comprehensive framework integrating thermal, economic, and spatial efficiency aspects for nano-insulation materials such as aerogels remains underdeveloped particularly in Middle Eastern contexts like Syria, where climatic and economic diversity strongly affect material performance.

Building upon previous work by Dory et al^42^. employing Degree-Day analysis for thermal and environmental assessment, the present study expands the scope to include multi-regional Syrian data and multiple fuel types (diesel, LPG, electricity). This research thus introduces an integrated tripartite framework thermal economic environmental to quantify how the unique nano-porous structure of aerogels alters both economic feasibility and ecological performance compared with conventional insulation materials.

## Materials and methods

### Aerogel

**Fig. 1:**
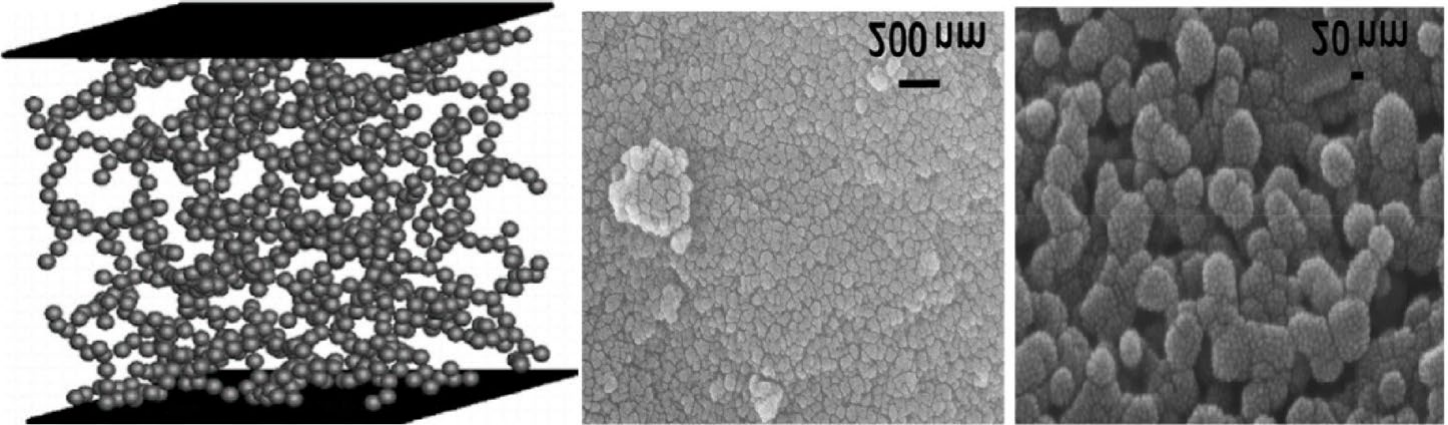
Nanostructure and Microscopic Characterization of the Highly Porous, Continuous 3D Aerogel^43,84^.

Nanotechnology has fundamentally advanced the development of high-performance thermal insulation materials, particularly through the introduction of aerogels^21^. Silica aerogels are among of the most extensively utilized nanoscale insulating materials, distinguished by their exceptional properties as the lightest known solid insulation material. These materials exhibit a transparent and homogeneous structure comprising a three-dimensional, interconnected nanoporous network (Fig. 1). This morphology originates from aggregation of silica particles into a highly porous microscopic matrix containing voids with diameters ranging from 2 to 50 nanometers^43–45^.

This nanoporous configuration superior multifunctional properties to aerogels, including:

- Extremely low density (typically 3–350 kg/m³) ^2^attributable to an ultra-high porosity approaching 99%^46,47^.
- Large specific surface area (≥ 1000 m²/g)^46,47^, which enhances adsorption and diffusion processes.
- Very low thermal conductivity ranging from 0.01 to 0.02 W/m·K^41^, average ≈ 0.013 W/m·K, markedly lower than that of conventional insulating materials^2^.
- Excellent acoustic insulation properties^48^, characterized by a low sound velocity (≈ 100 m/s)^45–47^.
- High optical transmittance exceeding 90–99% within specific wavelength ranges, enabling their use in transparent insulating panels^46,47^.
- Outstanding flame and combustion resistance, attributed to their inorganic silica composition^2^.
- Low dielectric constant (1.0–2.0)^46,47^, enabling applications in electronic insulation.
- Low refractive index (∼1.05)^46,47^, advantageous for optical and photonic applications.

These unique characteristics have stimulated extensive research and industrial interest in applying aerogels as innovative, high-efficiency insulating materials in the construction sector, including roofs, façades, windows, and energy-efficient envelope systems^45,49,50^.

### Studied insulation materials

Three conventional thermal insulation materials commonly used in Syrian construction systematically evaluated in comparison with nano-structured silica aerogel.

Table 1 lists the thermophysical properties of each material-density (ρ) and thermal conductivity (k_ins_)-and their median market prices in Syria during the survey period from December 2024 to June 2025. The materials selected in these assemblies consider local availability, cost-effectiveness, and established performance related to residential and commercial applications. On the other hand, silica aerogel is a new nano-insulation material with ultra-low thermal conductivity and high porosity, hence serving as an ideal benchmark for advanced thermal analysis.

**Table 1:** Thermophysical properties and cost per unit volume for each type of studied insulation materials^2,29,41,51^.

Thermal Insulation material	Density ρ (kg/m^3^)	Thermal Conductivity k_ins_ (W/m.K)	Cost C_ins_ ($/m^3^)
Extruded Polystyrene (XPS)	32	0.032	160
Glass Wool (GW)	22	0.04	70
Polyurethane (PUR)	35	0.028	345.1
Nano Aerogel	50	0.013	1400

### Methodology

#### Degree-day method

This study employs the Degree-Day mathematical framework to determine optimal insulation thickness for building walls. The heating and cooling Degree-Day (HDD/CDD) approach provides an efficient and reliable method for building energy assessment, based on the principle that thermal energy demand correlates directly with the temperature difference between outdoor air and a defined base temperature^21^^35^,. This method has been extensively validated in previous studies^52–55^–^56^.

The base temperature (Tb) represents the threshold of thermal neutrality the point at which mechanical heating or cooling becomes unnecessary to maintain indoor comfort^41^. For this analysis, benchmark base temperatures of Tb ≤ 15 °C and Tb > 24 °C were adopted for HDD and CDD calculations, respectively, across all investigated urban regions^57^.

The DD methodology conceptualizes building insulation as a capital investment requiring dual analysis of economic feasibility and environmental impact^58^. Previous research has determined optimal insulation thickness using the DD framework through multiple applications, including: heating load analyses^18^^30^^31^^33^^34^^54^^55^,,,,,,, cooling load assessments^18^^59–62^,–^63^, and comprehensive evaluations combined annual heating and cooling loads^18^^21^^52^^53^^64^,,,,. This method offers the practical advantage of requiring minimal input data while maintaining high predictive accuracy for simple building systems and applications^65^.

#### Study area and climatic data

**Fig. 2:**
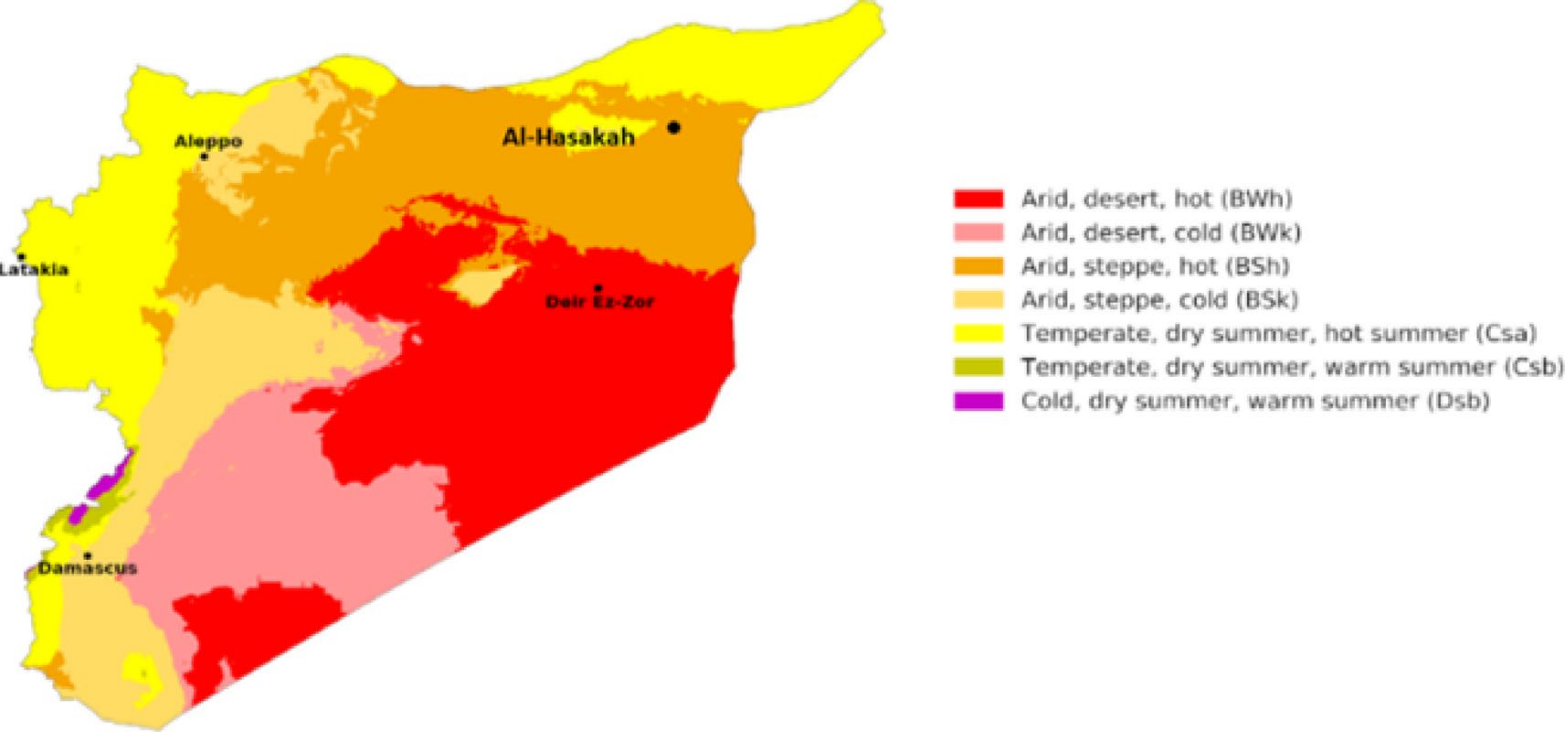
Köppen–Geiger climate classification map for Syria (1980–2016)^85^.

Thermal load assessments for both summer and winter conditions were conditions based.

on the Köppen–Geiger climate classification map^66^ for Syria, as illustrated in Fig. 2. This climatic framework categorizes Syria into distinct zones, with major urban centers distributed across four primary climate regions: (1) Arid, desert, hot (BWh), (2) Arid, steppe, hot (BSh), (3) Temperate, dry summer, hot summer (Csa), and (4) Arid, steppe, cold (BSk). To ensure comprehensive geographical representation, five urban centers were strategically selected: Deir Ezzor (Region 1), Al-Hasakah (Region 2), Latakia (Region 3), as well as Damascus and Aleppo (both representing Region 4). Climatic datasets including annual mean temperature, relative humidity and Degree-day (HDD/CDD) were collected for each region for the 2005–2023 period from the Photovoltaic Geographical Information System (PVGIS)^67^, maintained by the European Commission’s Joint Research Centre (JRC). The compiled data are presented in Table 2.

**Table 2:** Climatic and geographic parameters of the assessed urban centers.

No.	Region	City	latitude (Degree)	Longitude (Degree)	Elevation (m)	Average temperature(ºC)	HDD (ºC/Year)	CDD (ºC/Year)
1	BWh	Deir Ezzor	35.333 N	40.15 E	211	21.82	913	1697
2	BSh	Al-Hasakah	36.205 N	40.639 E	295	21.23	1061	1652
3	Csa	Latakia	35.509 N	35.757 E	9	19.77	513	616
4	BSk	Damascus	33.516 N	36.274 E	700	17.55	1359	666
5	BSk	Aleppo	36.199 N	37.164 E	407	18.73	1178	888

#### Structure of the external walls and case study

**Fig. 3:**
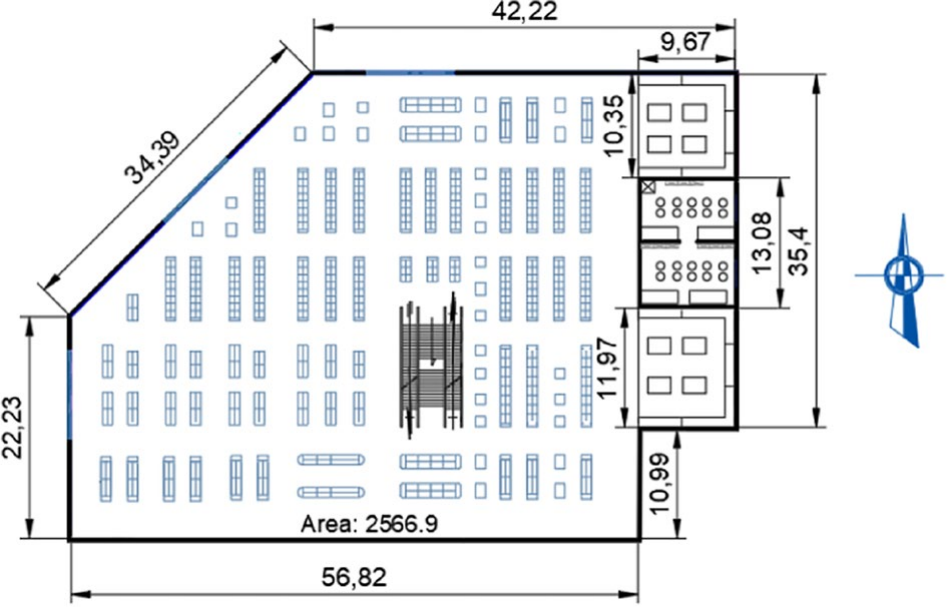
Floor plan of the studied commercial building.

**Fig. 4:**
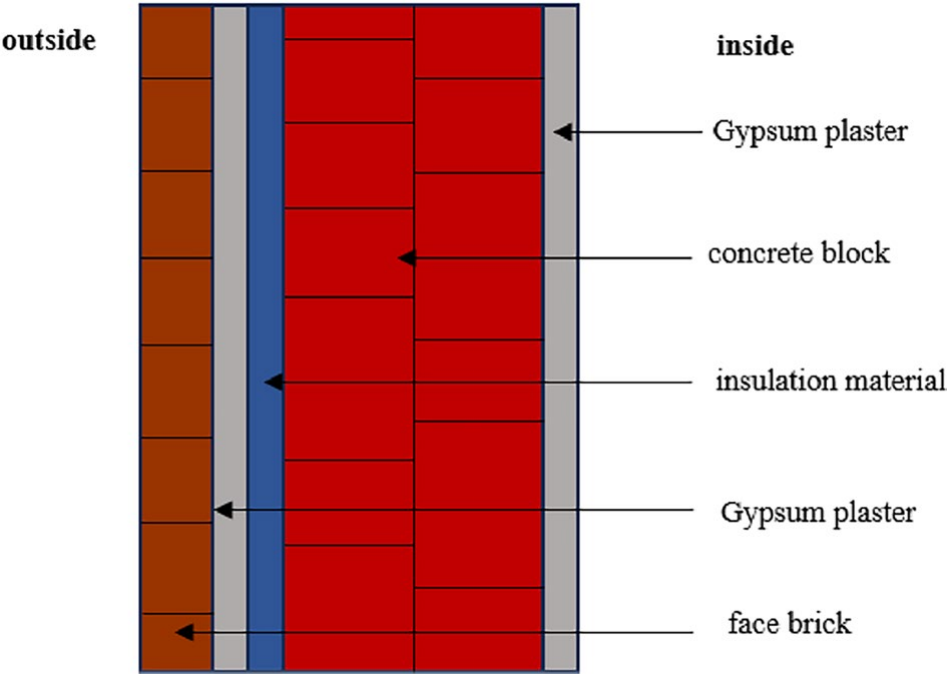
Structure of the studied external wall.

Thermal losses through building envelopes occur via four primary pathways: exterior walls, floor, roof, and fenestration systems, collectively accounting for approximately 15–35% of total building energy transfer^57^. This study focuses specifically on exterior wall assemblies, as they typically exhibit the highest heat flux density among envelope components. Consequently, external wall insulation serves as a critical strategy for enhancing overall building energy efficiency^68^, with potential savings of 50–60% achievable through optimized thermal insulation design^69^. The case study investigates a multi-layer external wall system in a 2500 m² commercial facility currently under construction (Fig. 3). The wall assembly consists of the following layers (from exterior to interior): 25-mm face brick, 12.7-mm gypsum plaster (outer coating), 200-mm concrete block (main structural layer), and 12.7-mm gypsum plaster (inner coating). A schematic cross-section of the wall structure is presented in Fig. 4, while the complete set of thermophysical properties (density, thickness, and thermal Resistance) for each layer is summarized in Table 3.

**Table 3:** Material properties of external Wall.

Wall Layers	Density (kg/m^3^)	Thickness (m)	Thermal Resistance (m^2^.K/W)	Total Resistance *R*_wt_ (m^2^.K/W)	U-value (W/m^2^.K)
R outside	-	-	0.05864	0.8344	1.198
Face brick	2002.3	0.025	0.01876		
Gypsum plaster	720.8	0.0127	0.05645		
Insulation	ρ_ins_	x_ins_	k_ins_		
concrete block	608.7	0.2	0.52348		
Gypsum plaster	720.8	0.0127	0.05645		
R inside	-	-	0.12064		

#### Evaluated fuel characteristics

Three conventional energy sources (diesel, liquefied petroleum gas (LPG), and electricity) were systematically evaluated as representative fuels for Syria’s energy sector. A standardized life-cycle cost analysis (LCC) analytical framework was applied to determine optimal insulation thicknesses corresponding to each fuel type.

The analysis utilized a validated dataset comprising thermophysical parameters such as the fuel heating value (H_u_, J.kg^[-1^) and heating system efficiency (η_s_), in addition to economic indicators including fuel cost (C_f_, $·m⁻³). All market data for the study period were obtained from the Syrian Ministry of Petroleum and Mineral Resources and are summarized in Table 4. Within the LCC framework, diesel and LPG were considered for heating applications, whereas electricity was analyzed for cooling systems, reflecting the current energy consumption patterns in Syrian buildings.

**Table 4:** Characterizations of the investigated fuels^38,70,71^.

Fuel Type	Chemical formula	C_f_	H_u_	η_s_
Diesel (Mazut)	C_7.3125_ H_10.407_ O_0.04_ S_0.026_ N_0.02_	0.90752($/kg)	41.278*10^6^ (J/kg)	0.8
LPG	C_3.7_H_4.1_	2.3 ($/m^3^)	46.453*10^6^ (J/m^3^)	0.9
Electricity	-	0.09233 $/kwh	3.599*10^6^ J/kwh	0.99

#### Heating and cooling load for external wall

**Fig. 5:**
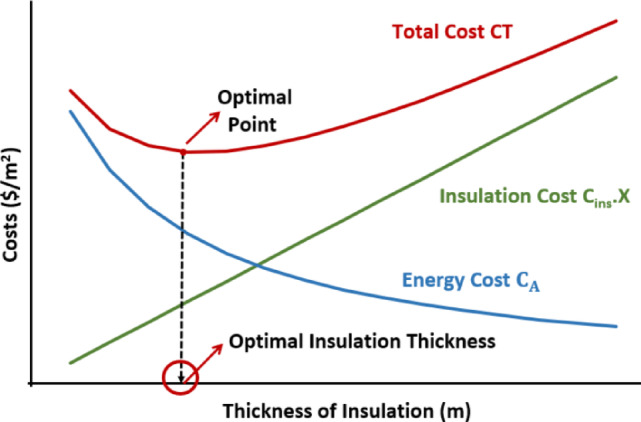
Conceptual schematic for the determination of optimal insulation thickness on the basis of minimum total cost.

The cost of insulation materials increases linearly with thickness, whereas the energy costs associated with heat transfer decrease exponentially. This inverse relationship necessitates a life-cycle cost analysis (LCC) to determine the economically optimal insulation thickness (Xₒₚₜ). The LCC methodology quantifies both net energy savings (Eₛ) and the Payback Period (Pb)^72^ by balancing the initial insulation investment material against long-term energy performance. The equilibrium point corresponds to the optimal insulation thickness, where total costs including both fuel consumption and insulation expenses are minimized, as illustrated in Fig. 5.

Mathematically, this relationship is described by a convex function that reaches its minimum at the economically optimal thickness^18^^[,27^^[,29^^[,73^. The determination of this optimal value follows the heating/cooling degree-day approach, as detailed below.

The annual thermal losses per unit wall area (q_A_) and the overall heat transfer coefficient (U) were determined using the following respective Eqs^38^^[,57^.

1 $$\:{\mathrm{q}}_{\mathrm{A}}=86400.\mathrm{D}\mathrm{D}.\mathrm{U}\:\:\:\:\:\:\:\:\:\:\:\:\:\:\:\:\:\:\:\:\:\:\:\:\:\:\:\:\:\:\:\:\:\:\:\:\:\:\:\:(\mathrm{J}/{\mathrm{m}}^{2}.\:\mathrm{y}\mathrm{e}\mathrm{a}\mathrm{r})$$

2 $$\:\mathrm{U}=\frac{1}{{\mathrm{R}}_{\mathrm{w}\mathrm{t}}+{\mathrm{R}}_{\mathrm{i}\mathrm{n}s}}\:\:=\frac{1}{{\mathrm{R}}_{\mathrm{w}\mathrm{t}}+\raisebox{1ex}{${\mathrm{X}}_{\mathrm{i}\mathrm{n}\mathrm{s}}$}\!\left/\:\!\raisebox{-1ex}{${\mathrm{K}}_{\mathrm{i}\mathrm{n}\mathrm{s}}$}\right.}\:\:\:\:\:\:\:\:(\mathrm{W}/{\mathrm{m}}^{2}.\mathrm{K})$$

Where, R_wt_, R_in_, X_ins_ and k_ins_​ represent the total thermal resistance, thermal resistance of the insulation layer, insulation thickness, and thermal conductivity of the insulation material, respectively.

Based on the Degree-day method, the annual energy consumption for heating (E_H_) and cooling (E_C_) was estimated by dividing the annual thermal losses (q_A_) by the system efficiency as follows^18,22,41,72,74^:

3 $$\:{\mathrm{E}}_{\mathrm{A},\mathrm{H}}=\frac{{\mathrm{q}}_{\mathrm{A},\mathrm{H}}}{{{\upeta\:}}_{s}}=\frac{86400.\mathrm{H}\mathrm{D}\mathrm{D}\:}{\left(\mathrm{R}\mathrm{w}\mathrm{t}+\raisebox{1ex}{$\mathrm{X}$}\!\left/\:\!\raisebox{-1ex}{$\mathrm{K}$}\right.\right).{{\upeta\:}}_{s}}\:\:\:\:\:\:\:\:\:\:\:\:\:\:\:\:\:\:(\mathrm{J}/{\mathrm{m}}^{2}.\:\mathrm{y}\mathrm{e}\mathrm{a}\mathrm{r})$$

4 $$\:{\mathrm{E}}_{\mathrm{A},\mathrm{C}}=\frac{{\mathrm{q}}_{\mathrm{A},\mathrm{C}}}{\mathrm{C}\mathrm{O}\mathrm{P}}=\frac{86400.\mathrm{C}\mathrm{D}\mathrm{D}\:}{\left(\mathrm{R}\mathrm{w}\mathrm{t}+\raisebox{1ex}{$\mathrm{X}$}\!\left/\:\!\raisebox{-1ex}{$\mathrm{K}$}\right.\right)\:.\mathrm{C}\mathrm{O}\mathrm{P}}\:\:\:\:\:\:\:\:\:(\mathrm{J}/{\mathrm{m}}^{2}.\:\mathrm{y}\mathrm{e}\mathrm{a}\mathrm{r})$$

The mentioned Coefficient of performance in cooling (COP), which depends on various operating parameters, was assumed to have an average value of 2.5 in this study^41^^[,52^^[,57^^[,72^^74,75^^76^..

Equation (5) provides the mathematical formulation for the annual fuel consumption rate (mfa) using the yearly heating transmission load^1^^[,57^,

5 $$\:\:\:\:\mathrm{m}\mathrm{f}\mathrm{a}=\frac{86400.\mathrm{H}\mathrm{D}\mathrm{D}}{\left({\mathrm{R}}_{\mathrm{w}\mathrm{t}}+\raisebox{1ex}{$\mathrm{X}$}\!\left/\:\!\raisebox{-1ex}{$\mathrm{K}$}\right.\right).{\mathrm{H}}_{\mathrm{u}}.{{\upeta\:}}_{S}}\:\:\:\:\:\:\:\:\:\:\:\:\:\:\:\:\:\:\:\:\:\:\:\:\:(\mathrm{k}\mathrm{g}/{\mathrm{m}}^{2}.\mathrm{y}\mathrm{e}\mathrm{a}\mathrm{r})$$.

Furthermore, the annual heating and cooling costs per unit area (C_A, H_, C_A, c​)_were derived from Eqs. (6) and (7), respectively^1^^[,57^,

6 $$\:\:\:\:\:\:\:\:{\mathrm{C}}_{\mathrm{A},\mathrm{H}}\:=\frac{86400.\mathrm{H}\mathrm{D}\mathrm{D}.{\mathrm{C}}_{\mathrm{f}}}{\left({\mathrm{R}}_{\mathrm{w}\mathrm{t}}+\raisebox{1ex}{$\mathrm{X}$}\!\left/\:\!\raisebox{-1ex}{$\mathrm{K}$}\right.\right){\mathrm{H}}_{\mathrm{u}}.{{\upeta\:}}_{S}}\:\:=\mathrm{m}\mathrm{f}\mathrm{a}.{\mathrm{C}}_{\mathrm{f}}\:\:\:\:(\mathrm{\$}/{\mathrm{m}}^{2})$$

7 $$\:\:\:\:\:\:\:\:{\mathrm{C}}_{\mathrm{A},\mathrm{C}}\:=\frac{86400.\mathrm{C}\mathrm{D}\mathrm{D}.{\mathrm{C}}_{\mathrm{e}}}{\left({\mathrm{R}}_{\mathrm{w}\mathrm{t}}+\raisebox{1ex}{$\mathrm{X}$}\!\left/\:\!\raisebox{-1ex}{$\mathrm{K}$}\right.\right)\mathrm{C}\mathrm{O}\mathrm{P}}\:\:\:\:=\mathrm{m}\mathrm{f}\mathrm{a}.{\mathrm{C}}_{\mathrm{e}}\:\:\:\:\:\:\:(\mathrm{\$}/{\mathrm{m}}^{2})$$.

Where mfa: Annual mass of fuel (kg/m^2^) or annual electricity consumption (kWh/m^2^).

Here, the specific electricity cost parameter (Cₑ) quantifies the per-unit energy cost for operating cooling systems that rely on electrical power. Finally, the total annual cost of energy was calculated as follows: ^1^

8 $$\:{\mathrm{C}}_{\mathrm{A}}={\mathrm{C}}_{\mathrm{A},\mathrm{H}}+{\mathrm{C}}_{\mathrm{A},\mathrm{C}}\:\:\:\:\:\:\:\:\:\:\:\:\:\:\:\:\:\:\:\:\:\:\:\:\:\:\:\:\:\:\:\:\:\:\:\:\:\:\:\:\:\:\:\:\:\:\:\:\:\:\:\:\:\:\:\:\:\:\:\:\:\:\:\:(\mathrm{\$}/{\mathrm{m}}^{2})$$

#### Life cycle cost

Scholarly investigations have proposed several financial methodologies for optimizing exterior wall insulation thickness. The Simple Payback Period approach, which estimates investments through operational savings, is limited by its neglect of the time value of money - a fundamental principle of financial analysis^18^.In contrast, Life Cycle Cost analysis (LCC) approach has emerged as the most comprehensive and reliable evaluation method^18^^25^^30^^77,78^,,,–^79^, providing a quantitative framework for assessing total system cost and.

insulation-derived energy savings over a 10-year operational period (*N* = 10 years)^25^^30^^31^^64^,,,.This method incorporates present-value conversion through the Present Worth Factor (PWF)^24^^29^^41^^57^^74^,,,,, which accounts for the real interest rate (r) derived from the nominal interest rate (g = 5.556%) and inflation rate (i = 8.25%). The real interest rate (r) is expressed as^1,22,31,33,41,57,72,74^:

9 $$\:\:\:\mathrm{r}=\frac{\mathrm{i}-\mathrm{g}}{1+\mathrm{g}}$$

The Present Worth Factor (PWF) over the system lifetime N is given by Eq. (10)^1,22,31,33,72,74^

10 $$\:\mathrm{P}\mathrm{W}\mathrm{F}=\frac{(1{+\mathrm{r})}^{\mathrm{N}}-1}{\mathrm{r}(1+{\mathrm{r})}^{\mathrm{N}}}$$

The total heating cost (CT_H_) and cooling cost (CT_C_) for an insulated wall under Life Cycle Cost (LCC) analysis are determined by Eqs. (11), (12), respectively^41^^[,57^.

11 $${\mathrm{C}}{{\mathrm{T}}_{\mathrm{H}}} = {{\mathrm{C}}_{{\mathrm{A}},{\mathrm{H}}}}.{\mathrm{PWF}} + {{\mathrm{C}}_{{\mathrm{ins}}}}.{\mathrm{X\;\;\;\;\;\;\;\;\;\;\;\;\;\;\;\;\;\;\;\;\;\;\;\;\;\;\;\;\;\;\;\;\;\;\;\;\;\;\;\;\;\;\;\;}}\left( {{{\$ }}/{{\mathrm{m}}^2}} \right)$$

12 $${\mathrm{C}}{{\mathrm{T}}_{\mathrm{C}}} = {{\mathrm{C}}_{{\mathrm{A}},{\mathrm{C}}}}.{\mathrm{PWF}} + {{\mathrm{C}}_{{\mathrm{ins}}}}.{\mathrm{X\;\;\;\;\;\;\;\;\;\;\;\;\;\;\;\;\;\;\;\;\;\;\;\;\;\;\;\;\;\;\;\;\;\;\;\;\;\;\;\;\;\;\;}}\;\;\left( {\$ /{{\mathrm{m}}^2}} \right)$$

Where: X is the insulation material thickness(m) and C_ins_ is the insulation material cost per unit volume ($/m^3^).

Neglecting additional installation and maintenance costs of insulation, the total life-cycle cost (CT) is defined as the sum of the total heating and cooling costs, given by^1,80^:.

13 $${\mathrm{CT}} = {\mathrm{PWF}}.{{\mathrm{C}}_{\mathrm{A}}} + {\mathrm{\;}}{{\mathrm{C}}_{{\mathrm{ins}}}}{\mathrm{\;}}.{\mathrm{X\;\;\;\;\;\;\;\;\;\;\;\;\;\;\;\;\;\;\;\;\;\;\;\;\;\;\;\;\;\;\;\;\;\;\;\;\;\;\;\;\;\;\;\;\;\;\;\;\;\;\;}}\left( {{{\$ }}/{{\mathrm{m}}^2}} \right)$$

14 $${\mathrm{\;CT}} = {\mathrm{PWF}}\left( {{{\mathrm{C}}_{{\mathrm{A}},{\mathrm{H}}}} + {{\mathrm{C}}_{{\mathrm{A}},{\mathrm{C}}}}\;} \right) + {\mathrm{\;}}{{\mathrm{C}}_{{\mathrm{ins}}}}{\mathrm{\;}}.{\mathrm{X\;\;\;\;\;\;\;\;\;\;\;\;\;\;\;\;\;\;\;\;\;\;\;\;\;\;\;\;\;\;\;}}\;\left( {\$ /{{\mathrm{m}}^2}} \right)$$

15 $${\mathrm{CT}} = {\mathrm{PWF}}\left( {{\mathrm{\;}}\frac{{{{\mathrm{q}}_{{\mathrm{A}},{\mathrm{H}}}}.{{\mathrm{C}}_{\mathrm{f}}}}}{{{{\mathrm{H}}_{\mathrm{u}}}.{{{\eta }}_S}}} + \frac{{{{\mathrm{q}}_{{\mathrm{A}},{\mathrm{c}}}}.{{\mathrm{C}}_{\mathrm{e}}}}}{{3.599 \times {{10}^6}.{\mathrm{COP}}}}{\mathrm{\;}}} \right) + {\mathrm{\;}}{{\mathrm{C}}_{{\mathrm{ins}}}}{\mathrm{\;}}.{\mathrm{X\;\;\;\;\;\;\;\;\;\;\;\;\;\;}}\left( {{{\$ }}/{{\mathrm{m}}^2}} \right)$$

The optimal insulation thickness is determined by minimizing the total life-cycle cost (CT) in Eq. (13). This is achieved by differentiating (CT) with respect to insulation thickness (X) and setting the result equal to zero. Consequently, the optimal insulation thickness for heating (X_opt, H_) and cooling (X_opt, c_)is obtained using Eqs. (16) and (17)^22,31,57,72^.

16 $$\:{\mathrm{X}}_{\mathrm{o}\mathrm{p}\mathrm{t},\mathrm{H}\:}=293.94\times\:{\left(\frac{\mathrm{H}\mathrm{D}\mathrm{D}\times\:\mathrm{C}\mathrm{f}\times\:\mathrm{P}\mathrm{W}\mathrm{F}\times\:{\mathrm{k}}_{\mathrm{i}\mathrm{n}\mathrm{s}}}{{\mathrm{H}}_{\mathrm{u}}\times\:{\mathrm{C}}_{\mathrm{i}\mathrm{n}\mathrm{s}}\times\:{{\upeta\:}}_{S}}\right)}^{\frac{1}{2}}-{\mathrm{k}}_{\mathrm{i}\mathrm{n}\mathrm{s}}\times\:{\mathrm{R}}_{\mathrm{w}\mathrm{t}}\:\:\:\:\:\:\left(\mathrm{m}\right)$$

17 $$\:{\mathrm{X}}_{\mathrm{o}\mathrm{p}\mathrm{t},\mathrm{C}}=293.94\times\:{\left(\frac{\mathrm{C}\mathrm{D}\mathrm{D}\times\:\mathrm{C}\mathrm{e}\times\:\mathrm{P}\mathrm{W}\mathrm{F}\times\:{\mathrm{k}}_{\mathrm{i}\mathrm{n}\mathrm{s}}}{{\mathrm{C}}_{\mathrm{i}\mathrm{n}\mathrm{s}}\times\:\mathrm{C}\mathrm{O}\mathrm{P}}\right)}^{\frac{1}{2}}-{\mathrm{k}}_{\mathrm{i}\mathrm{n}\mathrm{s}}\times\:{\mathrm{R}}_{\mathrm{w}\mathrm{t}}\:\:\:\:\:\:\:\:\:\:\left(\mathrm{m}\right)$$

The optimal insulation thickness that minimizes the total heating and cooling costs is derived as follows^18^:

18 $$\:{\mathrm{X}}_{\mathrm{o}\mathrm{p}\mathrm{t}}={\left(\frac{86400.\mathrm{P}\mathrm{W}\mathrm{F}.({\mathrm{C}}_{\mathrm{f}}.\mathrm{H}\mathrm{D}\mathrm{D}/{\mathrm{H}}_{\mathrm{u}}.{{\upeta\:}}_{S}+{\mathrm{C}}_{\mathrm{e}}.\mathrm{C}\mathrm{D}\mathrm{D}/\mathrm{C}\mathrm{O}\mathrm{P}){\mathrm{k}}_{\mathrm{i}\mathrm{n}\mathrm{s}}}{{\mathrm{C}}_{\mathrm{i}\mathrm{n}\mathrm{s}}}\right)}^{\frac{1}{2}}-{\mathrm{R}}_{\mathrm{w}\mathrm{t}}.\:{\mathrm{k}}_{\mathrm{i}\mathrm{n}\mathrm{s}}\:\:\:\:\:\:\left(\mathrm{m}\right)$$

##### Life cycle cost savings and discounted payback period

Life cycle cost savings (LCS) term is annual net savings achieved through the use of insulation materials. It is calculated as the difference between the total energy cost savings accumulated over the system lifetime and the initial investment of insulation^81^, as defined follows^31^^72^,

19 $$\:\:\:\:\:\:\:\:\mathrm{L}\mathrm{C}\mathrm{S}={\mathrm{C}}_{\mathrm{A}\:,\mathrm{P}\mathrm{r}\mathrm{e}\:\mathrm{i}\mathrm{n}\mathrm{s}}-\mathrm{C}\mathrm{T}\:\:\:\:\:\:\:\:\:\:\:\:\:\:\:\:\:\:\:\:\:\:\:\:\:\:\:\:\:\:\:\:\:\:\:\:\:\:\:\:\:\:\:\:\:\:\:\:\:\:\:\:\:\:\:\:\:\:\:\:\:\:\:\:\:\:\:\:\:\:\:(\mathrm{\$}/{\mathrm{m}}^{2})$$

20 $$\:\mathrm{L}\mathrm{C}\mathrm{S}={\mathrm{C}}_{\mathrm{A}\:,\mathrm{P}\mathrm{r}\mathrm{e}\:\mathrm{i}\mathrm{n}\mathrm{s}}.\mathrm{P}\mathrm{W}\mathrm{F}-{\mathrm{C}}_{\mathrm{A}}.\mathrm{P}\mathrm{W}\mathrm{F}-{\mathrm{C}}_{\mathrm{i}\mathrm{n}\mathrm{s}}.\mathrm{X}\:\:\:\:\:\:\:\:\:\:\:\:\:\:\:\:\:\:\:\:\:\:\:\:\:\:\:\:\:\:\:\:\:(\mathrm{\$}/{\mathrm{m}}^{2})$$.

The expression (C_A, pre ins_.PWF-C_A_.PWF) represents the energy cost savings per unit wall area, derived from the difference between non-insulated and insulated building states. Consequently, the economic viability of insulation systems was further assessed using the Discounted payback period (DPB), defined as the time required for cumulative energy savings to offset the initial insulation investment^22,72^:

21 $$\:\mathrm{D}\mathrm{P}\mathrm{B}=\frac{{\mathrm{C}}_{\mathrm{i}\mathrm{n}\mathrm{s}}.X}{\mathrm{L}\mathrm{C}\mathrm{S}}\:\:\:\:\:\:\:\:\:\:\:\:\:\:\:\:\:\:\:\:\:\:\:\:\:\:\:\:\:\:\:\:\:\:\:\:\:\:\:\:\:\:\:\:\:\:\:\:\:\:\:\:\:\:\:\:\:\:\:\:\:\:\:\:\:\:\:\:\:\:\:\:\:\:\:\:\:\:\:\:\:\:\:\:\:\:\:\:\:\:\:\:\:\:\:\:\:\left(\mathrm{y}\mathrm{e}\mathrm{a}\mathrm{r}\right)$$

#### Ecological emissions analysis

The continuous increase in the global population intensifies energy demands, which remains predominantly met through fossil fuel consumption. This dependency generates substantial greenhouse gas (GHG) emissions and harmful atmospheric pollutants. Enhancing the thickness and efficiency of building insulation materials significantly mitigates these environmental impacts by reducing heat transfer through the building envelope, thereby lowering the energy requirements for heating and cooling systems. This reduction in energy consumption directly decreases fuel usage and consequently diminishes the associated air pollution (CO₂ and SO₂.). The general chemical equation for hydrocarbon fuel combustion can be expressed as follows^57^^73^^[,82^,,

22 $$\:{C}_{x}+{H}_{z}+{O}_{w}+{S}_{y}+{N}_{t}+\alpha\:.A({O}_{2}+3.76{N}_{2})\to\:x{CO}_{2}+\left(\frac{z}{2}\right){H}_{2}O+y{SO}_{2}+B.{O}_{2}+E.{N}_{2}$$.

The constants A and E are determined from the oxygen balance formulas as follows^1^^82^,,

23 $$\:A=(x+y+\frac{z}{4}-\frac{w}{2})$$

24 $$\:\mathrm{E}=3.76{\upalpha\:}(\mathrm{x}+\mathrm{y}+\frac{\mathrm{z}}{4}-\frac{\mathrm{w}}{2})+\frac{\mathrm{t}}{2}$$.

Consistent with Eq. (29), CO and NOₓ emissions are excluded. Emission rates for dominant combustion byproducts (CO₂ and SO₂) are computed per kilogram of combusted fuel using Eqs. (25) and (26)^1^^[,57^^[,82^,

25 $$\:\mathrm{m}\mathrm{C}{O}_{2}=\frac{\mathrm{x}\:\mathrm{C}{\mathrm{O}}_{2}}{\mathrm{M}}\:\:\:\:\:\:\:\:\:\:\:\:\:\:\:\:\:\:\:\:\:\:\:\:\:\:\:\:\:\:\:\:\:\:\:\:\:\:\:\:\:\:\:\:\:\:\:\:\:\:\:\:\:\:\:\:\:\:\:\:(\mathrm{k}\mathrm{g}\mathrm{C}{\mathrm{O}}_{2}/\mathrm{k}\mathrm{g}\:\mathrm{f}\mathrm{u}\mathrm{e}\mathrm{l})$$

26 $$\:\mathrm{m}\mathrm{S}{\mathrm{O}}_{2}=\frac{\mathrm{y}\:\mathrm{S}{\mathrm{o}}_{2}}{\mathrm{M}}\:\:\:\:\:\:\:\:\:\:\:\:\:\:\:\:\:\:\:\:\:\:\:\:\:\:\:\:\:\:\:\:\:\:\:\:\:\:\:\:\:\:\:\:\:\:\:\:\:\:\:\:\:\:\:\:\:\:\:\:(\mathrm{k}\mathrm{g}\mathrm{S}{\mathrm{O}}_{2}/\mathrm{k}\mathrm{g}\:\mathrm{f}\mathrm{u}\mathrm{e}\mathrm{l})$$.

The molar mass of fuel M kg·kmol⁻¹ was calculated as^1,57,82^

27 $$\:M = 12x + z + 16w + 32y + 14t\:\:\:\:\:\:\:\:\:\:\:\:\:\:\:\:\:\:\:\:\:\:({\mathrm{kg}} \cdot {\mathrm{kmo}}{{\mathrm{l}}^{ - 1}})$$

Consequently, the annual CO₂ and SO₂ emissions are calculated from the fuel consumption rate (mfa) using Eqs. (28) and (29)^1^^[,57^^[,82^,

28 $$\:\mathrm{m}\:\mathrm{C}{\mathrm{O}}_{2}=\frac{44\:\mathrm{x}}{\mathrm{M}}\:\mathrm{m}\mathrm{f}\mathrm{a}\:\:\:\:\:\:\:\:\:\:\:\:\:\:\:\:\:\:\:\:\:\:\:\:\:\:\:\:\:\:\:\:\:\:\:\:\:\:\:\:\:\:\:\:\:\:\:\:\:\:(\mathrm{k}\mathrm{g}/{\mathrm{m}}^{2}.\mathrm{y}\mathrm{e}\mathrm{a}\mathrm{r})$$

29 $$\:\mathrm{m}\:\mathrm{S}{\mathrm{O}}_{2}=\frac{64\:\mathrm{y}}{\mathrm{M}}\:\mathrm{m}\mathrm{f}\mathrm{a}\:\:\:\:\:\:\:\:\:\:\:\:\:\:\:\:\:\:\:\:\:\:\:\:\:\:\:\:\:\:\:\:\:\:\:\:\:\:\:\:\:\:\:\:\:\:\:\:\:\:(\mathrm{k}\mathrm{g}/{\mathrm{m}}^{2}.\mathrm{y}\mathrm{e}\mathrm{a}\mathrm{r})$$.

## Results and discussion

### Optimal insulation thickness

**Fig. 6:**
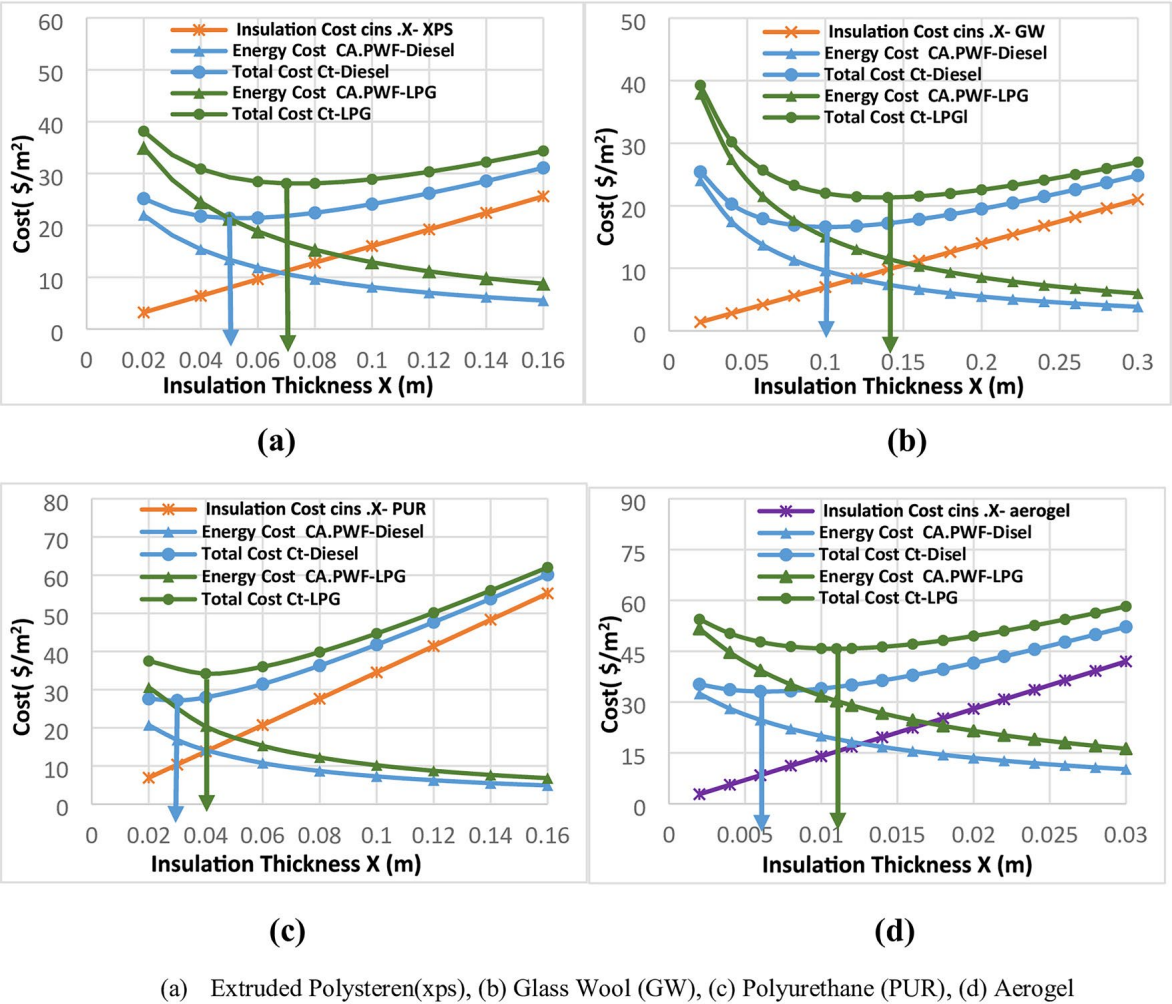
optimal insulation thickness for Deir Ezzor region for all investigated insulation materials: (**a**) Extruded Polysteren(xps), (**b**) Glass Wool (GW), (**c**) Polyurethane (PUR), (**d**)Aerogel.

This study presents a comprehensive comparative analysis of four insulation materials (extruded polystyrene (XPS), glass wool (GW), polyurethane (PUR), and nano-silica aerogel) to evaluate their optimal thickness, energy savings, and economic performance across five distinct Syrian climatic zones (Deir Ezzor, Al-Hasakah, Latakia, Damascus, and Aleppo). The analysis considers three energy sources: diesel and liquefied petroleum gas (LPG) for heating, and electricity for cooling applications. Figure 6 illustrates the relationship between insulation thickness and total cost components (energy cost, insulation cost, and combined cost) for external walls over a 10-year life-cycle period across all examined regions. The primary purpose of increasing insulation thickness in buildings is to minimize heat transfer losses through the envelope. directly reduces heating and cooling energy demands thereby lowering overall fuel consumption and associated operating costs. However, this reduction in energy expenditure is counterbalanced by a linear increase in material costs as insulation thickness grows. The total cost, defined as the sum of insulation material expenses and discounted lifetime energy costs, exhibits a convex optimization behavior. It initially decreases with increasing thickness as energy losses are reduced, reaches a minimum at the economically optimal thickness, and then rises again beyond this point as the incremental cost of additional insulation outweighs further energy savings. This convex relationship underpins the life-cycle cost (LCC) methodology applied in this study, enabling the determination of the most cost-effective insulation thickness that ensures both thermal efficiency and economic sustainability across diverse climatic conditions.

**Fig. 7:**
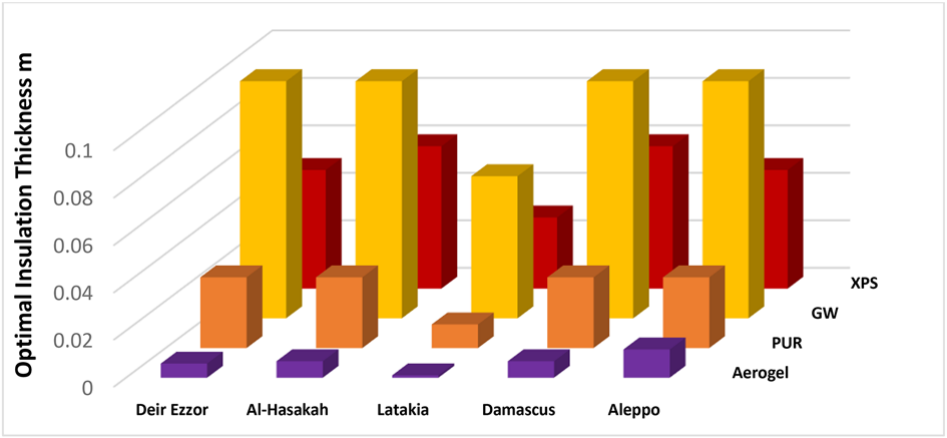
Optimal insulation thickness for various materials and regions utilizing diesel fuel.

**Fig. 8:**
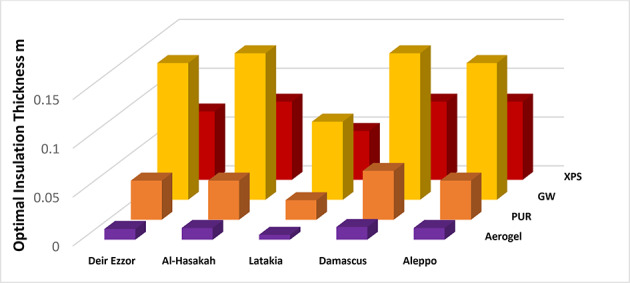
Optimal insulation thickness for various materials and regions utilizing LPG fuel.

Figures 7 and 8 present the calculated optimum insulation thickness values corresponding to different fuel types diesel and liquefied petroleum gas (LPG) and insulation materials across the selected climatic regions. For diesel fuel, glass wool (GW) exhibited the highest optimum insulation thickness, with values ranging from 0.06 m to 0.1 m followed by extruded polystyrene (XPS) ranging from 0.03 m to 0.06 m, and Polyurethane (PUR) ranging from 0.01 m to 0.03 m. In contrast, aerogel consistently achieved the lowest optimum thickness among all insulation materials, ranging from 0.001 m to 0.006 m, which consistent with the inverse relationship between an insulation material’s thermal conductivity and its optimal thickness, as well established in the literature^35^.

This corresponds to a remarkable thickness reduction approximately 94–98.3.3% compared with Glass Wool, 90–96.6.6% compared with XPS, and 80–90% compared with PUR. Such superior performance is attributed to exceptionally low thermal conductivity of aerogel, which results from its highly nanoporous structure and minimal solid conduction pathways.

Similarly, when LPG fuel was considered, aerogel again demonstrated significant superiority in terms of optimal insulation thickness, with values ranging between 0.005 m and 0.01 m. Aerogels achieved thickness reduction approximately 91.33–93.75%, 83.75–90.75%, and 74–75% compared to GW, XPS, and PUR, respectively. previously studied traditional insulation materials, respectively. These results further confirm the.

outstanding thermal efficiency of aerogel, highlighting its potential to minimize insulation.

volume while maintaining or exceeding the energy performance of traditional materials.

The comparative analysis revealed consistently higher optimal insulation thickness values for all insulation materials when using liquefied petroleum gas compared to diesel fuel, resulting in a smaller relative reduction in optimal thickness for aerogel under LPG use. However, hierarchy of materials performance remained unchanged across both fuels, following the inverse relationship between thermal conductivity and optimal thickness for each insulation type. However, the hierarchy of material performance remained unchanged across both fuels, following the inverse relationship between thermal conductivity and optimal thickness for each insulation type. This variation is primarily attributed to the relative cost differential favoring LPG over diesel, which enhances the economic feasibility of investing in higher-efficiency insulation systems.

Regarding the studied climatic zones, the analysis of diesel-based heating revealed remarkable similarities in optimal insulation thickness between Aleppo and Deir ez-Zor on one hand, and between Al-Hasakah and Damascus on the other. In contrast, Latakia exhibited a distinct deviation, showing reduced optimal thickness values of 83.3–85.71.3.71% for aerogel, 66.6% for PUR, (40–50) % for XPS, and 40% for GW compared to the average of other zones.

When LPG was used, a similar pattern was observed, except for the Latakia again demonstrated a clear reduction 54.54–61.53% for aerogel, (50–60) % for PUR, 42.85–46.66) % forb GW, and 28.57–37.5% for XPS This deviation stems from the lower heating and cooling degree-day (HDD/CDD) values characteristic of Latakia’s coastal climate, confirming that optimal insulation thickness is highly sensitive to climatic variability, particularly between temperate coastal and continental interior zones.

These results highlight the necessity of developing climate-specific insulation standards rather than adopting uniform national guidelines, especially in countries like Syria, where regional climatic contrasts are substantial.

Table 5 summarizes the optimal insulation thickness, energy cost savings, total life-cycle costs, life-cycle savings, and Discounted payback period all evaluated cases prior to accounting for space efficiency considerations. Among all tested materials, glass wool demonstrated the best overall economic performance, achieving the highest energy cost savings (ES = 11.86–60.6$/m²), the lowest total life-cycle costs (CT = 10.8–23.6 $/m²). Furthermore, it achieved the most favorable life-cycle savings (LCS) (7.66–50.1$/m²) and the shortest Discounted payback period (DPB = 2.1–5.5 years). However, these advantages were accompanied by the greatest insulation thickness requirements, which may reduce usable interior space and diminish potential rental revenue in commercial settings.

In contrast, aerogel, despite its exceptionally low thickness (0.001–0.01 m) and superior thermal efficiency, exhibited the least favorable economic performance before accounting for spatial benefits, due to its high initial cost. Aerogel recorded the lowest energy savings (ES = 1.56–40.2 $/m²), the highest total life-cycle cost (CT = 18.3–51.75 $/m²), the lowest life-cycle savings (LCS = 0.16–22 $/m²), and the longest Discounted payback period (DPB = 8.3–88.8 years). Nevertheless, its ultra-thin structure offers a distinct advantage in applications where spatial efficiency is critical, such as high-value urban real estate, building retrofits, and commercial complexes, where maximizing floor area directly translates to higher rental profitability. Extruded polystyrene and polyurethane demonstrated intermediate performance relative to glass wool and aerogel, achieving balanced energy savings and life-cycle cost, with moderate Discounted Payback Periods ranging from 3 to 7.9 years for XPS and 4.4–16.6 years for PUR. The adoption of LPG fuel resulted in greater energy cost savings compared to diesel, thereby reducing total life-cycle costs and shortening of Discounted payback period across all materials and climatic zones.

This finding positions LPG as an economically advantageous heating fuel, yet contrasts with Dombayci et al.^25^, who identified coal as the most cost-effective energy source in Denizli, Turkey. The discrepancy arises from differing economic parameters, particularly the ratio $\:{\mathrm{C}}_{\mathrm{f}}/{\mathrm{H}}_{\mathrm{u}}.{{\upeta\:}}_{\mathrm{S}}$ which defines the cost of usable energy.

In the present study, the specific combination of a higher effective fuel price (C_f_) combined with lower calorific value (H_u_) and system efficiency $\:\left({{\upeta\:}}_{S}\right)$ yielded a higher energy cost ratio, thereby increasing the financial benefit of insulation.

Conversely, coal’s lower cost per energy unit in the Turkish context made it more economical without extensive insulation. This contrast underscores the importance of localized, context-specific energy and insulation policies, a principle particularly vital in post-reconstruction and energy-efficiency strategies for Syria. Continental interior zones recorded shorter Discounted Payback Period, attributed to higher thermal load intensities (cooling/heating), that necessitate greater insulation thickness to achieve energy efficiency. In contrast, the coastal region (Latakia) recorded longer Discounted payback period due to moderate thermal loads. These results confirm that the economic performance of insulation improves with increasing heating-day (HDD) and cooling-day (CDD) values, as higher thermal demands yield greater energy savings and accelerate recovery of the initial investment. This outcome aligns with Fertelli et al.^38^ who reported a similar trend across Turkey’s climatic zones, with optimal insulation thickness from warmer regions (e.g., Aydın: 1213 HDD) to colder ones (e.g., Sivas: 3444 HDD) and shorter Discounted Payback Periods in colder climates. This agreement between these findings and previous literature reinforces the universal relevance of climate-adapted insulation strategies, particularly in countries with diverse climates conditions such as Syria.

**Table 5:** Optimal insulation thickness, Energy savings cost, life cycle cost, life cycle cost savings, and for the investigated cases.

Fuel type	Insulation materials	Optimal thickness X_opt_(m)	Energy savings Cost ($/m^2^)	CT ($/m^2^)	LCS ($/m^2^)	DPB (years)
Deir Ezzor						
Diesel	Without insulation			-	-	-
	XPS	0.05	25.04	21.37	17.04	4.7
	GW	0.1	28.8	16.61	21.8	3.2
	PUR	0.03	21.59	27.17	11.24	9.2
	Aerogel	0.006	13.68	33.13	5.28	15.9
LPG	Without insulation			-	-	-
	XPS	0.07	44.25	28.08	33.05	3.4
	GW	0.14	49.6	21.33	39.8	2.5
	PUR	0.04	40.74	34.2	26.93	5.1
	Aerogel	0.011	30.78	45.75	15.38	10
Al-Hasakah						
Diesel	Without insulation			-	-	-
	XPS	0.06	28.84	22.43	19.24	5
	GW	0.1	31.24	17.43	24.24	2.9
	PUR	0.03	23.43	28.6	13.07	7.9
	Aerogel	0.007	16.34	35.13	6.54	15
LPG	Without insulation			-	-	-
	XPS	0.08	51.04	29.83	38.24	3.3
	GW	0.15	55.94	22.64	45.44	2.3
	PUR	0.04	45.36	36.51	31.56	4.4
	Aerogel	0.012	35.75	49.12	18.95	8.9
Latakia						
Diesel	Without insulation			-	-	-
	XPS	0.03	9.76	13.49	4.96	9.7
	GW	0.06	11.86	10.8	7.66	5.5
	PUR	0.01	5.53	16.37	2.08	16.6
	Aerogel	0.001	1.56	18.3	0.16	88.8
LPG	Without insulation			-	-	-
	XPS	0.05	20.35	18.87	12.35	6.5
	GW	0.08	22.19	14.63	16.59	3.4
	PUR	0.02	15.6	22.52	8.7	7.9
	Aerogel	0.005	9.85	28.37	2.85	24.6
Damascus						
Diesel	Without insulation			-	-	-
	XPS	0.06	27.63	21.9	18.03	5.3
	GW	0.1	29.94	16.99	22.94	3.1
	PUR	0.03	22.45	27.84	12.1	8.6
	Aerogel	0.007	15.66	34.07	5.86	16.7
LPG	Without insulation			-	-	-
	XPS	0.08	55.29	31.25	42.49	3
	GW	0.15	60.6	23.65	50.1	2.1
	PUR	0.05	52.66	38.35	35.4	4.9
	Aerogel	0.013	40.2	51.75	22	8.3
Aleppo						
Diesel	Without insulation			-	-	-
	XPS	0.05	24.44	21.05	16.44	4.9
	GW	0.1	28.11	16.38	21.11	3.3
	PUR	0.03	21.08	26.77	10.73	9.7
	Aerogel	0.006	13.35	32.54	4.95	17
LPG	Without insulation			-	-	-
	XPS	0.08	50.09	29.52	37.29	3.4
	GW	0.14	54.21	22.4	44.41	2.2
	PUR	0.04	44.52	36.09	30.71	4.5
	Aerogel	0.012	35.09	48.52	18.29	9.2

Although glass wool exhibited the best overall insulation performance across all evaluated cases when spatial considerations were excluded (Table 5), its significant thickness requirement (ranging from 0.03 m to 0.15 m; see Figs. 7 and 8) reduces usable interior floor area negatively impacts potential rental revenue. Consequently, determining the most economically viable insulation solution strategy necessitates incorporating spatial efficiency metrics and their influence on rental income into both life-cycle cost and discounted payback period analyses.

The average annual rental rate for commercial properties, expressed per square meter (USD/m²·year), is summarized in Table 6 for five major Syrian cities representing distinct climatic and economic conditions: Deir Ezzor, Al-Hasakah, Latakia, Damascus, and Aleppo. These data provide the basis for quantifying the economic value of space savings resulting from reduced insulation thickness, particularly when comparing conventional materials (e.g., GW, XPS, PUR) with advanced nano-insulation materials such as aerogel.

**Table 6:** Rent cost of different studied regions^83^.

City	Rent cost ($/m^2^. year)
Deir Ezzor	140
Al-Hasakah	165
Latakia	200
Damascus	400
Aleppo	300

This comprehensive evaluation, summarized in Table 7 directly compares the optimal thickness performance of glass wool (acting as a baseline reference where spatial considerations were excluded) against nano-aerogel and other conventional insulation alternatives. The comparison integrates spatial efficiency indicators by incorporating area savings, life-cycle savings (LCS), and discounted payback period (DPB) into the assessment (for all other studied materials). The results reveal a consistent pattern: aerogel systematically achieves the minimum optimal thickness and the maximum space preservation across all evaluated cases. These findings not only reinforce the foundational principles established by Ali et al.^41^, but also extend their applicability across diverse climatic and economic contexts, confirming that nano-insulation technologies enable substantial material reduction while maximizing spatial utility relative to conventional materials.

Contextual analysis based on the Life Cycle Cost (LCC) assessment for Damascus revealed a significant economic advantage for aerogel insulation, which produced the highest net savings of 75.04 $/m² with LPG and 41.94 $/m² with diesel. This outcome is primarily from aerogel’s superior thermal efficiency and ultra-low optimal insulation thickness which resulted in the largest gain in usable interior space 31.6 m² (LPG) and 21.5 m² (diesel).

**Fig. 9:**
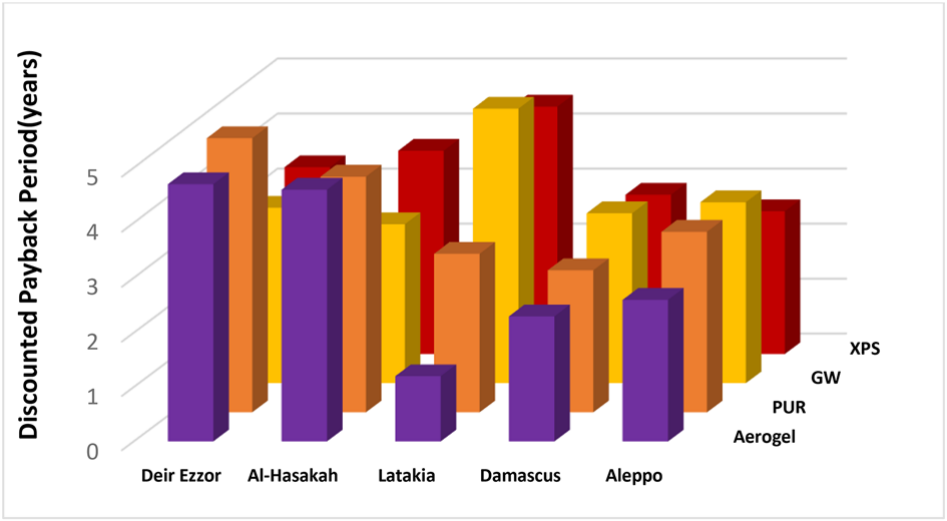
Discounted payback period for insulation materials across various regions utilizing Diesel fuel.

**Fig. 10:**
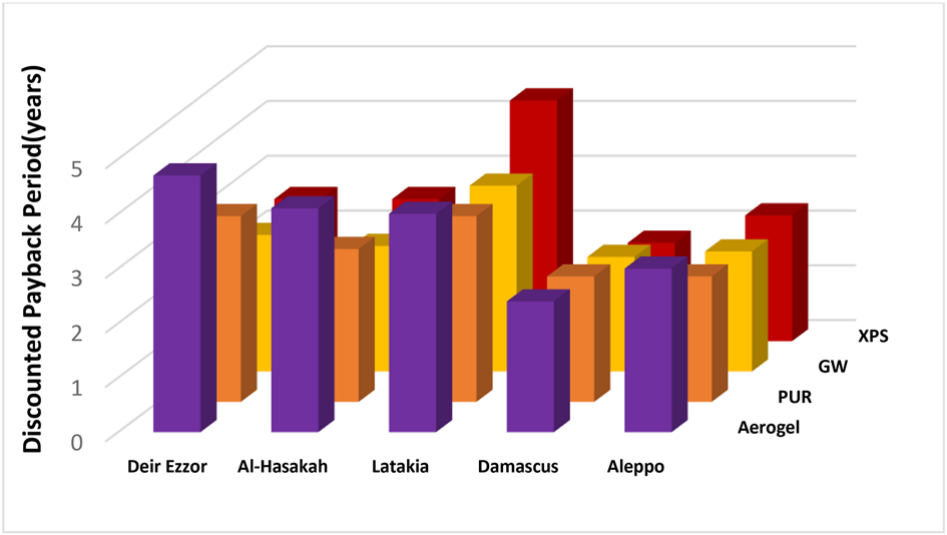
Discounted payback period for insulation materials across various regions utilizing LPG fuel.

The economic impact of this saved area was further amplified by the high commercial rental rates in Damascus, a critical parameter in the LCC model that significantly amplified the overall net savings. From an investment perspective, aerogel achieved the shortest Discounted payback period with diesel (2.3 years; Fig. 9) and a slightly longer yet still competitive value with LPG (2.4 years) compared with the of 1.8–2.3.8.3-year range observed for the other materials (Fig. 10).

A similar trend was observed in Aleppo, where aerogel delivered high life-cycle savings 55.42 $/m² for LPG and 32.26 $/m² for diesel with the shortest Discounted payback period with diesel 2.6 years and a competitive value for LPG (3 years for vs. 2.3 years for other materials).

This strong performance is attributed to Aleppo’s relatively high rental rates and considerable space savings (29.5 m² for LPG and 21.7 m² for diesel). Aerogel also exhibited superior performance in Latakia under diesel heating, achieving the shortest Discounted payback period of 1.2 years, significantly lower than the 2.9–4.5 years observed for other materials. It also yielded net savings of 11.58 $/m², comparable to the 10.76–11.73$/m² range for alternative materials. The significant reduction in the discounted payback period, from 80.8 years to 1.2 years, is directly attributed to the remarkably low optimal thickness (0.001 m) achieved through the optimization process. This minimal thickness resulted in a very low initial investment cost (C_in_=0.001 × 1400 = 1.4$/m^2^), which was rapidly offset by the high economic value of the recovered floor space. Consequently, the lower the optimal thickness, the more dominant the space-saving benefits become in shortening the Discounted payback period, as the marginal cost of the material becomes negligible compared to the rental or real estate value of the reclaimed area.

However, with LPG, aerogel showed an average Discounted payback period of 4 years, comparable to 3.4–4.4 years for other materials, but registered the lowest net savings of 17.36 $/m² versus 18.15–20.37 $/m² among all insulation types. Regarding the remaining study areas (Deir Ezzor and Al-Hasakah), aerogel maintained its superior space-saving advantage but exhibited the longest Discounted Payback Periods and lowest net savings exhibited to XPS and PUR. This outcome is primarily attributed to the low rental values in these regions (Table 6), which diminished the economic impact of aerogel’s spatial advantage. Among conventional materials, extruded polystyrene (XPS) demonstrated notable economic superiority in Deir Ezzor, yielding the shortest recovery times (3.4 years for diesel and 2.6 years for LPG) and the highest net savings of 23.8 $/m² and 42.53 $/m² for diesel and LPG, respectively. A similar pattern was found in AL-Hasakah, where XPS exhibited Discounted Payback Periods between 2.6 and 3.7 years and net savings between 25.62 $/m² and 49.41 $/m² for diesel and LPG respectively. Although XPS achieved lower space savings than aerogel 11.5–16.1 m² in Deir Ezzor and 9.2–16.1 m² in Al-Hasakah, its superior economic performance is explained by the higher ambient temperatures and cooling loads in these zones (Table 2), which necessitate greater insulation thicknesses (0.05–0.07 m in Deir Ezzor and 0.06–0.08 m in Al-Hasakah). In such context, XPS provides sufficient thermal efficiency at moderate cost, representing a cost-effective solution due to its unit price (≈ 160 $/m³). This cost advantage is particularly relevant in regions with lower rental rates, where the economic gain from spatial efficiency becomes less influential in determining the most viable insulation choice.

**Table 7:** Life-cycle cost analysis and Discounted payback period of the Investigated insulation materials incorporating the space optimization benefits.

Fuel type	Insulation materials	Optimal thickness X_opt_(m)	Area savings (m^2^)	LCS ($/m^2^)	DPB (years)
Deir Ezzor					
Diesel	XPS	0.05	11.5	23.8	3.4
	GW	0.1	-	21.8	3.2
	PUR	0.03	16.2	20.76	5
	Aerogel	0.006	21.7	18.02	4.7
LPG	XPS	0.07	16.1	42.53	2.6
	GW	0.14	-	39.8	2.5
	PUR	0.04	23.1	40.51	3.4
	Aerogel	0.011	29.8	32.88	4.7
Al-Hasakah					
Diesel	XPS	0.06	9.2	25.62	3.7
	GW	0.1	-	24.24	2.9
	PUR	0.03	16.2	24.3	4.3
	Aerogel	0.007	21.5	21.43	4.6
LPG	XPS	0.08	16.1	49.41	2.6
	GW	0.15	-	45.44	2.3
	PUR	0.04	25.4	49.16	2.8
	Aerogel	0.012	31.8	40.97	4.1
Latakia					
Diesel	XPS	0.03	6.9	10.76	4.5
	GW	0.06	-	7.66	5.5
	PUR	0.01	11.5	11.73	2.9
	Aerogel	0.001	13.6	11.58	1.2
LPG	XPS	0.05	6.9	18.15	4.4
	GW	0.08	-	16.59	3.4
	PUR	0.02	13.9	20.37	3.4
	Aerogel	0.005	17.3	17.36	4
Damascus					
Diesel	XPS	0.06	9.2	33.5	2.9
	GW	0.1	-	22.94	3.1
	PUR	0.03	16.2	39.3	2.6
	Aerogel	0.007	21.5	41.94	2.3
LPG	XPS	0.08	16.1	69.58	1.8
	GW	0.15	-	50.1	2.1
	PUR	0.05	23	74.05	2.3
	Aerogel	0.013	31.6	75.04	2.4
Aleppo					
Diesel	XPS	0.05	11.5	30.94	2.6
	GW	0.1	-	21.11	3.3
	PUR	0.03	16.2	31.13	3.3
	Aerogel	0.006	21.7	32.26	2.6
LPG	XPS	0.08	13.8	54.7	2.3
	GW	0.14	-	44.41	2.2
	PUR	0.04	23.1	59.82	2.3
	Aerogel	0.012	29.5	55.42	3

Polyurethane (PUR) demonstrated notable economic advantages in Latakia when using LPG, achieving the shortest discounted payback period (DPB = 3.4 years) and highest net savings 20.37 $/m². Under diesel operation, PUR also recorded the highest net diesel savings 11.73$/m² with an average discounted payback period of 2.9 years. A similar trend was observed in Aleppo, where PUR achieved the highest net savings (59.82 $/m²) and the shortest discounted payback period (DPB = 2.3 years) under LPG conditions.

This strong economic performance is attributed to the balanced thermal efficiency of PUR and the moderate optimal thickness among the studied materials. For example, in Aleppo, the optimal thickness of PUR was 0.04 m, compared to 0.08 m for XPS and 0.012 m for aerogel under LPG usage. These moderate characteristics resulted in acceptable space savings; 13.9 m² with LPG, 11.5 m² with diesel in Latakia, and 23.1 m² with LPG in Aleppo. Although these space savings were lower than those achieved by aerogel, PUR represents a more cost-effective solution due to the lower initial cost (≈ 345.1 $/m³).

Additionally, the larger optimal thickness of XPS relative to that of PUR led to smaller space savings, thereby enhancing the relative economic attractiveness of PUR in coastal regions. In Latakia, the moderate rental value 200 $/m²·year and manageable insulation requirements, consistent with the local heating and cooling degree -days, further supported the feasibility of PUR, particularly when paired with LPG’s high combustion efficiency. In Aleppo, despite higher commercial rental rates (≈ 300 $/m²·year), the intermediate thickness and moderate cost of PUR enabled favorable net savings and short Discounted Payback Periods, amplified by the material’s strong thermal performance under LPG operation. Overall, PUR exhibited average performance relative to XPS and aerogel across the remaining climatic zones, offering a balanced compromise between space utilization, energy savings, and economic return.

Collectively, this study confirms the economic viability of nano-silica aerogel as an advanced insulation material that effectively balances superior thermal performance with favorable financial returns, particularly in urban areas characterized by high real estate values and in coastal regions predominantly reliant on diesel fuel. The distinctive advantage of aerogel lies in its unparalleled ability to maximize usable interior space, thereby generating financial gains that offset its higher initial cost while simultaneously enhancing overall building sustainability. The strong economic performance of aerogel in high-rental cities directly corroborates the findings of Ali et al.^41^, emphasizing the pivotal role of spatially driven economic returns as a key determinant in the feasibility of nano-insulation. applications. Conversely, conventional materials such as extruded polystyrene and polyurethane remain competitive and contextually advantageous in specific climatic or fuel-dependent scenarios, reinforcing the necessity for localized, data-driven insulation material selection frameworks. This research underscores the essential need to integrate spatial efficiency and property valuation metrics comprehensive insulation performance material assessments, thereby advancing the analytical paradigm beyond traditional energy-centric evaluation models. Such an integrated techno economic spatial approach represents a crucial step toward developing sustainable, climate-responsive building insulation strategies tailored to the heterogeneous urban and climatic conditions of countries such as Syria.

### Environmental emissions analysis

**Fig. 11:**
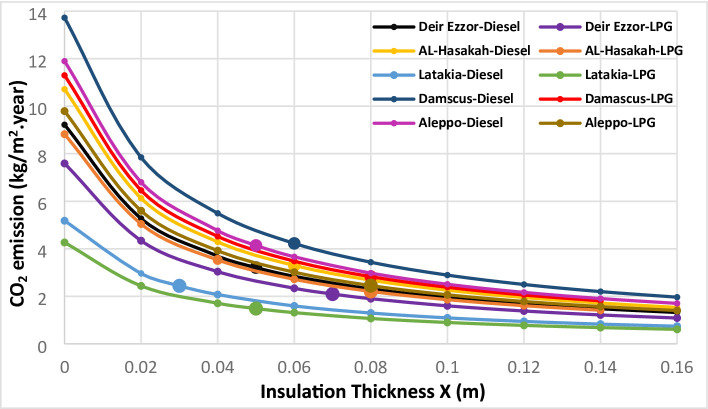
CO_2_ emissions of Extruded Polystere (XPS) under diverse climatic conditions and fuel types.

**Fig. 12:**
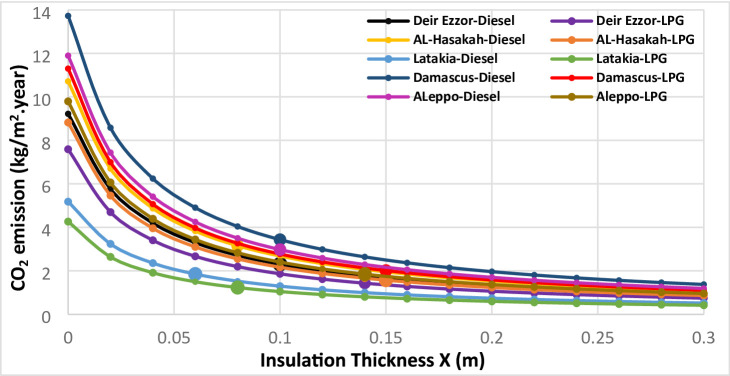
.CO_2_ emissions of Glass Wool (GW) under diverse climatic conditions and fuel types.

**Fig. 13:**
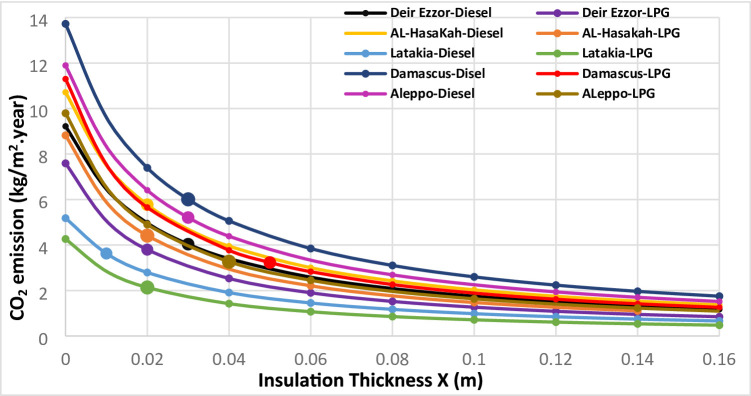
CO_2_ emissions of Polyurethane (PUR)under diverse climatic conditions and fuel types.

**Fig. 14:**
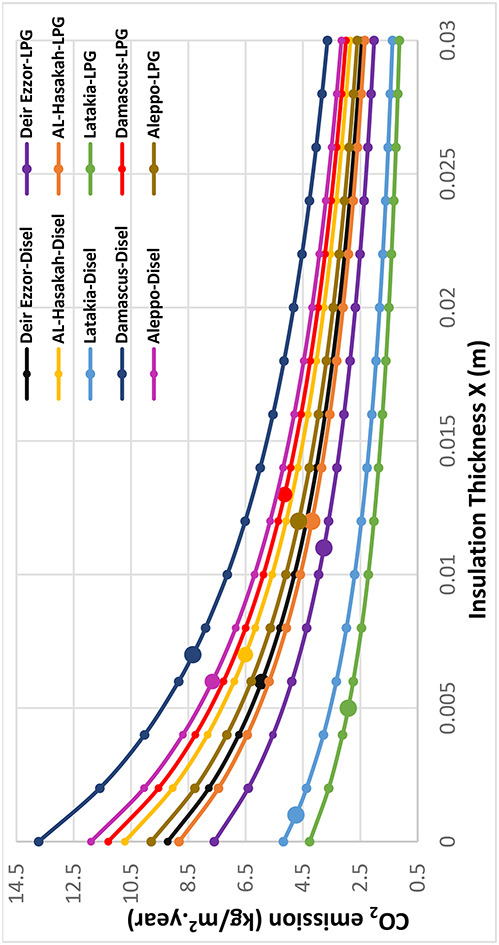
CO_2_ emissions of Aerogel under diverse climatic conditions and fuel types.

**Fig. 15:**
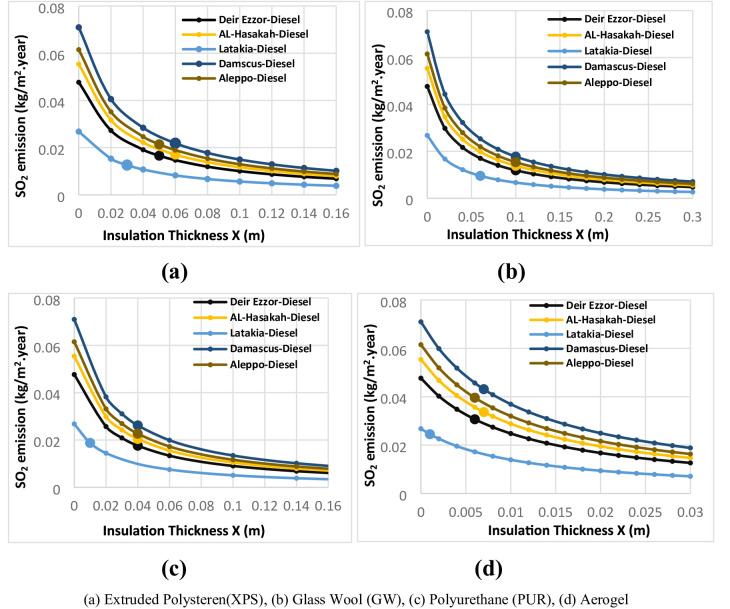
SO2 emissions Utilizing LPG fuel under diverse climatic conditions and all investigated insulation materials: (**a**) Extruded Polysteren(XPS), (**b**) Glass Wool (GW), (**c**) Polyurethane (PUR), (**d**) Aerogel.

The environmental impacts associated with different thermal insulation materials were evaluated across multiple Syrian climate zones. The assessment considered diesel and liquefied petroleum gas (LPG) for heating applications, and electricity for cooling systems. Using a simplified static approach based on design-day heating and cooling temperatures, the annual thermal load was computed to estimate corresponding greenhouse gas emissions. Figures 11, 12, 13, 14 and 15 illustrate the relationship between insulation thickness and annual CO₂ and SO₂ emissions for various cities and fuel types.

The findings reveal a clear inverse correlation between insulation thickness and the emission levels of both CO₂ and SO₂. a trend consistently reported in previous studies^73^.

The emissions curves exhibit a sharp decline at smaller insulation thicknesses, followed by a progressively slower decrease as thickness increases, indicating diminishing marginal efficiency of emission reduction at higher insulation levels. Furthermore, emission rates demonstrated a strong inverse correlation with the thermal conductivity of the evaluated materials across all thicknesses. Glass wool, characterized by the highest thermal conductivity, consistently yielded greatest CO₂ and SO₂ emissions, whereas aerogel, with its ultra-low thermal conductivity, achieved the lowest emission levels across all case studies. This outcome confirms that thermal conductivity (k) is the dominant parameter governing emission-reduction efficiency, validating the critical role of material nano-structure in minimizing environmental impacts^73^.

summarizes the fuel consumption and corresponding CO₂ and SO₂ emissions at the optimal insulation thickness for each material and fuel type. The implementation of optimal insulation thicknesses revealed substantial variations in environmental performance among the studied materials. Due to its relatively high optimal thickness, G.W exhibited the lowest fuel consumption rate for all scenarios, ranging from 0.37 to 1.07 kg/m² year. Consequently, G.W also yielded the lowest emission levels, with CO₂ emissions between 1.23–3.43 kg/m²·year and SO₂ emissions between 0.01–0.02 kg/m²·year, corresponding to an emission reduction efficiency of 64.26–82.17%. In contrast, aerogel showed the highest fuel consumption **(**1.12–2.59 kg/m². year), resulting in the highest emission levels (CO_2_: 3.77–8.34 kg/m². year; SO_2_: 0.01–0.04 kg/m². year). The emission reduction rate for aerogel only from 8.44% to 52.52%, indicating a comparatively limited environmental performance relative to conventional materials. Among the other materials, XPS s extruded polystyrene (XPS) demonstrated superior environmental performance compared with polyurethane (PUR). XPS achieved an emission reduction rate of 65.19% to 74.98%, with CO_2_ and SO_2_ emissions in the ranges of 1.48–4.23 kg/m²·year and 0.01–0.02 kg/m²·year, respectively. In comparison, PUR exhibited lower reduction efficiency (29.97% to 66.64%) and higher emissions (CO₂: 2.13–6.00 kg/m². year; SO₂: 0.02–0.03 kg/m². year). This finding, where the material with higher thermal conductivity (XPS) achieved greater emission reduction than that with lower conductivity (aerogel), contrasts with earlier studies^36^. the pivotal role of economic parameters, particularly insulation material cost and life-cycle optimization, in determining the overall environmental impact of thermal insulation applications. It is also noteworthy that when diesel fuel was used, CO₂ emissions consistently exceeded SO₂ emissions by a factor of approximately 150–270 across all cases (Table 8). This disproportionate ratio is primarily attributed to the significantly higher carbon content relative to sulfur in diesel fuel composition, which inherently produces far more carbon dioxide than sulfur dioxide during combustion.

**Table 8:** Fuel consumption, gas emissions at the optimal insulation thickness for Diesel and LPG across selected Syrian cities.

Fuel type	Insulation materials	Optimal thickness X_opt_(m)	mfa (kg/m^2^. year)	Co_2_ emission (kg/m^2^.year)	So_2_ emission (kg/m^2^.year)
Deir Ezzor					
Diesel	Without insulation		2.86	9.22	0.05
	XPS	0.05	1	3.21	0.02
	GW	0.1	0.72	2.31	0.01
	PUR	0.03	1.25	4.04	0.02
	Aerogel	0.006	1.84	5.94	0.03
LPG	Without insulation		2.26	7.59	-
	XPS	0.07	0.62	2.1	-
	GW	0.14	0.43	1.43	-
	PUR	0.04	0.75	2.53	-
	Aerogel	0.011	1.12	3.77	-
Al-Hasakah					
Diesel	Without insulation		3.33	10.71	0.06
	XPS	0.06	1.02	3.3	0.02
	GW	0.1	0.83	2.68	0.01
	PUR	0.03	1.46	4.69	0.02
	Aerogel	0.007	2.02	6.51	0.03
LPG	Without insulation		2.63	8.82	-
	XPS	0.08	0.66	2.21	-
	GW	0.15	0.47	1.57	-
	PUR	0.04	0.88	2.94	-
	Aerogel	0.012	1.25	4.19	-
Latakia					
Diesel	Without insulation		1.61	5.18	0.03
	XPS	0.03	0.76	2.44	0.01
	GW	0.06	0.57	1.85	0.01
	PUR	0.01	1.13	3.63	0.02
	Aerogel	0.001	1.47	4.74	0.02
LPG	Without insulation		1.27	4.27	-
	XPS	0.05	0.44	1.48	-
	GW	0.08	0.37	1.23	-
	PUR	0.02	0.64	2.13	-
	Aerogel	0.005	0.87	2.92	-
Damascus					
Diesel	Without insulation		4.26	13.72	0.07
	XPS	0.06	1.31	4.23	0.02
	GW	0.1	1.07	3.43	0.02
	PUR	0.03	1.87	6	0.03
	Aerogel	0.007	2.59	8.34	0.04
LPG	Without insulation		3.37	11.3	-
	XPS	0.08	0.84	2.83	-
	GW	0.15	0.6	2.01	-
	PUR	0.05	0.96	3.23	-
	Aerogel	0.013	1.53	5.14	-
Aleppo					
Diesel	Without insulation		3.69	11.9	0.06
	XPS	0.05	1.29	4.14	0.02
	GW	0.1	0.92	2.98	0.02
	PUR	0.03	1.62	5.21	0.03
	Aerogel	0.006	2.38	7.66	0.04
LPG	Without insulation		2.92	9.79	-
	XPS	0.08	0.73	2.45	-
	GW	0.14	0.55	1.85	-
	PUR	0.04	0.97	3.27	-
	Aerogel	0.012	1.39	4.65	-

Beyond the intrinsic material properties, the geographical location and its corresponding climatic severity emerged as a dominant determinants of fuel consumption and emissions levels. A strong positive correlation was observed between the Heating Degree Days (HDD) and both fuel consumption and greenhouse gas emissions across all five investigated cities. To illustrate this relationship, two extreme cases were examined. Latakia, characterized by the lowest HDD value (513 °C·day), exhibited the lowest fuel consumption (0.37–1.47 kg/m². year) and consequently the lowest emission levels (CO₂: 1.23–4.74 kg/m². year; SO₂: 0.01–0.02 kg/m². year) corresponding to an emission reduction efficiency of 8.44–71.08%.

In contrast, Damascus, with the highest HDD (1359 °C), consistently produced the highest fuel consumption values, with a range of 0.6 to 2.59 kg/m². year. and the greatest CO₂ and SO₂ emissions CO₂: 2.01–8.42 kg/m²·year; SO₂: 0.02–0.04 kg/m²·year. Despite insulation application achieving a substantial relative emission reduction (39.22–82.17%) compared with uninsulated case, the absolute emission levels in Damascus remained the highest, underscoring the dominant influence of climatic demand on environmental performance. The remaining three cities exhibited intermediate results, proportionally aligned with their respective HDD values, confirming the overarching of climate effect on insulation related energy and emission outcomes. These results collectively demonstrate that while thermal insulation is a highly effective mitigation strategy, the total environmental impact is ultimately governed by local climatic intensity.

Fuel type also played a decisive role in emission outcomes.

Liquefied Petroleum Gas (LPG) consistently resulted in significantly lower carbon emissions compared with diesel across all scenarios, due to its lower carbon content. This aligns with the emphasis placed on energy source importance for emission reduction strategies^36^. This reduction effect was most pronounced in high-HDD cities such as Damascus and Aleppo. Material-specific analysis revealed that polyurethane (PUR) achieved the highest emission reduction (64.48%) in Aleppo under LPG operation, followed by glass wool (GW) in Damascus (41.35%) and extruded polystyrene (XPS) in Aleppo (40.81%). Conversely, aerogel exhibited the lowest emission improvement (39.30%) in Damascus, reflecting the combined influence of material cost, limited thickness, and fuel type. The demonstrated superiority of LPG over diesel becomes particularly significant when paired with conventional insulation materials, especially in regions with high heating demands. These comprehensive findings highlight a synergistic interaction between insulation material characteristics, geographical location, and fuel type, confirming that all three parameters are critical in optimizing the environmental and economic performance of thermal insulation systems.

## Conclusion

This study presented a comprehensive thermo-economic and environmental analysis and optimization of conventional and nano-scale insulation materials, namely glass wool (GW), extruded polystyrene (XPS), polyurethane (PUR), and aerogel, in buildings under various Syrian climatic conditions with diesel and liquefied petroleum gas (LPG) for heating and electricity for cooling. The results showed that the insulation performance is essentially controlled by the interaction of material properties, climatic severity, and local economic conditions. Aerogel had the smallest optimal thickness, in the range of 0.001–0.013 m, due to its ultra-low thermal conductivity and nanostructure; it had the lowest energy savings in the range of 1.56–40.2 $/m² and the highest life-cycle cost in the range of 18.3–51.75.3.75 $/m². On the contrary, glass wool necessitated the highest thickness, in the range of 0.06–0.15 m, while providing the highest energy saving in the range of 11.86–60.6 $/m² and the minimum total cost in the range of 10.8–23.6 $/m². XPS and PUR gave intermediate values. Aerogel yielded a thickness reduction of 94–98.3.3%, 90–96.6.6%, and 80–90% with respect to GW, XPS, and PUR, respectively, for diesel heating, while LPG gave slightly lower values.

In high-demand, high-rental cities such as Damascus and Aleppo, aerogel yielded the highest net life-cycle savings (75.04 $/m² with LPG) and the shortest Discounted payback period (1.2 years in Latakia using diesel). In contrast, conventional materials were more cost-effective in regions with moderate heating loads and lower rental values, such as Deir Ezzor and Al-Hasakah, where their lower initial costs outweighed the spatial advantages of aerogel.

From an environmental perspective, aerogel showed higher fuel consumption and emissions, achieving the lowest CO₂ reduction efficiency (8.44–52.52%) relative to uninsulated walls. In contrast, GW achieved the best environmental performance with CO₂ and SO₂ reduction rates of 64.26–82.17%, while XPS outperformed PUR in all emission-related indicators. These findings confirm that environmental performance is not solely determined by thermal conductivity, but by the overall life-cycle balance between cost, thickness, and thermal demand.

The climatic severity of the location, represented through Heating Degree Days, became the prime driver for both energy consumption and emissions. Damascus, with the highest HDD, had the largest absolute emission values in spite of quite high relative reductions, while Latakia had the lowest due to its mild coastal climate. In all the different scenarios, CO₂ emissions from diesel combustion were 150 to 270 times as high as SO₂ emissions, which was indicative of the higher C/S ratio of diesel. Regional carbon emissions were considerably reduced by the use of LPG, notably in the case of Damascus and Aleppo. The highest emissions reduction was brought about by PUR (64.48% in Aleppo with LPG), followed by GW in Damascus (41.35%) and XPS in Aleppo (40.81%), with aerogel having the poorest improvement (39.30% in Damascus).

In conclusion, while offering unmatched thermal performance and great space savings, the economic and environmental viability of aerogel depends on the context in which it is used. GW, XPS, and PUR retain their competitive or even preferable status in areas with low energy demand and construction costs. These findings point to a locally adapted, climate-sensitive framework, which incorporates thermal efficiency, life-cycle economics, and spatial performance into decisions on energy-efficient and sustainable building in Syria’s reconstruction strategy.

## Research limitations

In this study several limitations should be considered. Relying on the heating/cooling degree-day method, a simplified model, means that dynamic factors such as daily temperature variations and direct solar radiation are not captured, unlike in more complex dynamic simulation models. Additionally, the economic analysis is based on the assumption of constant prices for insulation materials and fuel over the ten-year study period, an assumption that does not reflect the severe economic volatility of the Syrian market, where price fluctuations could dramatically change key outcomes like the Discounted Payback Period. The environmental analysis focused on operational emissions (resulting from fuel combustion), without taking into account the full life cycle environmental impact (LCA) of the insulating materials themselves, such as embodied energy and carbon emissions during manufacturing, transportation, and disposal, which could potentially affect the results in some cases. Therefore, the findings are highly specific to the conditions defined in this study, including local climates, system efficiencies, and economic parameters used, and cannot be generalized absolutely to other contexts.

## Future works

The third direction aims to explore nano-engineered, sustainable insulation materials, emphasizing molecular- and micro-structural optimization to enhance thermal performance while maintaining cost-effectiveness and practical applicability in the local construction sector.

Building upon the findings and limitations of this study, future research will advance thermal insulation optimization in Syria through three complementary directions. The first direction involves applying and validating advanced dynamic simulation models supported by high-resolution local climate data to more accurately represent transient environmental conditions, including diurnal temperature fluctuations, solar radiation, and heat-storage dynamics. The second direction focuses on developing probabilistic economic frameworks that integrate stochastic fuel price modeling and full life cycle assessment (LCA) of insulation materials, enabling a more realistic evaluation of financial feasibility and environmental impacts under uncertainty. The third direction aims to explore nano- engineered of sustainable insulation emphasizing molecular structural optimization to enhance thermal performance while maintaining cost-effectiveness and practical applicability in the local construction sector.

Collectively, these research directions will contribute to establishing a holistic framework for insulation material selection that harmonizes technical performance, economic sustainability, and environmental responsibility. Such advancements will ultimately support the development of more resilient, energy-efficient building practices across Syria’s diverse climatic zones.

## Data Availability

The data used to support the findings of this study are available from the corresponding author upon request.

## Declarations

**All authors** reviewed and approved the final version of the manuscript.
